# Discovery of triazole derivatives for biofilm disruption, anti-inflammation and metal ion chelation

**DOI:** 10.3389/fchem.2025.1545259

**Published:** 2025-02-26

**Authors:** Shuang Hong, Hongzhi Lu, Dawei Tian, Yue Chang, Qi Lu, Feng Gao

**Affiliations:** Department of Pediatrics, Maternity and Child Health Hospital of Qinhuangdao, Qinhuangdao, China

**Keywords:** 1,2,4-triazole derivatives, anti biofilm, anti inflammatory, antiasthmatic, metal ion detection

## Abstract

In the face of bacterial hazards to human health and resistance to multiple antibiotics, there is an urgent need to develop new antibiotics to meet the challenge. In this paper, the triazolyl heterocyclic (3-amino-1,2,4-triazole, **D**) was synthesised efficiently using thiourea as starting material. Finally, the end product **E** was obtained by aldehyde-amine condensation reaction and the structures of all compounds were determined by spectral analysis. *In vitro* antimicrobial activity showed that **E10** had a MIC of 32 μg/mL against the tested *Escherichia coli* and 16 μg/mL against the tested *Staphylococcus aureus* strain. Meanwhile, **E10** has a good anti-biofilm effect. Antibacterial mechanism studies have shown that **E10** has a good membrane targeting ability, thus disrupting cell membranes, leading to leakage of intracellular proteins and DNA and accelerating bacterial death. In terms of anti-inflammation, **E10** dose-dependently inhibits the levels of inflammatory factors NO and IL-6, which deserves further exploration in the treatment of asthma. The study of metal ion removal capacity showed that the synthesised triazole derivatives have high capacity to remove heavy metals Pb^2+^, Cd^2+^, Ca^2+^, Mg^2+^, Fe^3+^,Cr^3+^ and Al^3+^ in the range of 42%–60%.

## 1 Introduction

The ESKAPE (*E. coli*/*Escherichia coli*, *S. aureus*/*Staphylococcus aureus*, *Klebsiella* pneumonia/*K. pneumoniae*, *Acinetobacter* Baumannii/A. baumannii, *Pseudomonas* aeroginosa/P. aeroginosa and *Enterobacter* species) pathogens pose an enormous health and economic threat to humans and the farming industry. These pathogens are resistant to drugs through a variety of mechanisms, rendering them invulnerable to human intervention (Ma et al., 2020; Jadimurthy et al., 2022; Patil et al., 2021). It has even been reported that bioepidermal infections account for about 2/3 of these infections (Wang et al., 2022). The emergence of multidrug-resistant (MDR), extensively drug-resistant (XDR) and pan-drug-resistant (PDR) bacteria triggered by conventional antibiotics has exacerbated the problem, and therefore there is an urgent need to develop new therapeutic approaches for the treatment of bacterial drug resistance and biofilm infections (Mulani et al., 2019; Rakesh et al., 2018).

**FIGURE 1:**
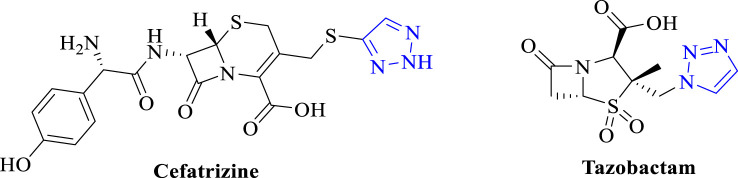
Chemical structure of triazole moiety in antibacterial.

Triazoles are common pharmacophores in many drugs and have important advantages in overcoming drug resistance, reducing toxicity and improving pharmacokinetics due to their unique molecular structure and mechanism of action (Zafar et al., 2021). Triazole derivatives have various pharmacological properties such as anti-tuberculosis (Zhang et al., 2017), anti-fungal (Dheer et al., 2017), and antibacterial (Fan et al., 2018). Triazoles can also act as stable linkage units, mimicking the electronic properties of amide bonds, carboxylic acids, etc., to link different pharmacophores (Li and Zhang, 2022). Schiff bases are an important class of organic compounds because they readily form stable complexes with most transition metals and play an important role in the development of coordination chemistry (Dalia et al., 2018). This complex has become a new type of antibacterial drug due to its better antibacterial activity and reduction of antibiotic resistance (Teran et al., 2019). Therefore, the combination of triazoles with Schiff bases is a sensible strategy in order to develop new effective drug candidates against bacterial threats (Matin et al., 2022). Therefore, the compounds designed in this paper are the couplings of triazole and Schiff bases to study their antimicrobial and anti-biofilm capabilities. As shown in Figure 1, several antimicrobial derivatives containing triazole structures have been used in clinical applications for the treatment of infections caused by a wide range of microorganisms.

Water quality is vital to human survival, as water is an essential element for life (Scorza et al., 2024). However, a major reason for the serious water pollution nowadays is the excessive heavy metal ions (Botle et al., 2023). Therefore, how to purify heavy metal ions in water is a difficult problem we face, and Schiff bases tend to have better metal chelating properties. More studies have reported that the antibacterial activity of Schiff base may be related to its complexing metals (Manhas et al., 2021). Schiff bases can chelate ions essential for microbial development or interact with DNA to cause irreversible damage to pathogens (Arag´on-Muriel et al., 2021). For these reasons, the ability of synthetic triazole derivatives coupled with Schiff bases to remove heavy metal ions from water was measured in this paper. We also know that infections are often accompanied by inflammation when they occur, and we also tested the anti-inflammatory effects of compounds.

In summary, in this paper, 3-amino-1,2,4-triazole (**D**) was synthesised using thiourea as a starting material, and finally the antimicrobial agent **E**, a coupling of triazoles and Schiff bases, was obtained by aldehyde-amine condensation reaction. The synthesised triazole derivatives have high removal rate of heavy metal ions and good antibacterial and anti-biofilm effect.

## 2 Results and discussion

### 2.1 Chemical synthesis

**SCHEME 1:**
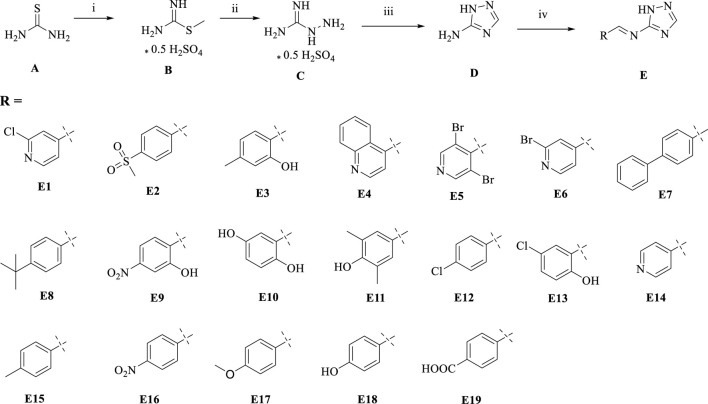
Synthesis of triazole derivatives. Conditions and reagents: (i) Methanol and CH_3_I, reflux, 12h, yield 93%; (ii) Ethanol, NH_2_NH_2_, reflux, yield 88%; (iii) Ethanol, HCOOH, reflux, yield 85%; (iV) Ethanol, different aldehyde groups, reflux, yield 50%–91%.

We used thiourea as a starting material in a three-step reaction to obtain the intermediate triazole derivative 3-amino-1,2,4-triazole (**D**) (Manning et al., 2002; Stojković et al., 2023). The reaction yields of the first three steps were above 85%, and finally the end product **E**, a coupling of triazoles and Schiff bases, was obtained by aldehyde-amine condensation reaction, and the synthetic route is showed in Scheme 1. All products were characterised by NMR and mass spectra.

### 2.2 The antibacterial activity of the compounds

#### 2.2.1 Determination of minimum inhibitory concentration

Some triazole derivatives have been reported to have good *in vitro* antibacterial activity (Tian et al., 2023). In this experiment, the *in vitro* inhibitory activity (MIC) of the Gram-positive bacteria *S. aureus* ATCC 29213, *S. aureus* ATCC 43300, *S. aureus* ATCC 33731, *S. aureus* MRSA2 and the Gram-negative bacteria *E. coli* ATCC 25922, *E*. *coli* DE 17 were determined using the micro broth dilution method. The antimicrobial results of the compounds are shown in Table 1. Among the tested compounds, **E10** showed good inhibitory activity against *E. coli* at a concentration of 32 μg/mL. In addition, compound **E10** showed inhibitory activity against all *S. aureus* strains at a concentration of 64 μg/mL. We found that the addition of hydroxyl group to the ligand benzaldehyde increased the antimicrobial activity by analysing the structure-activity relationship between compounds **E3**, **E9**, **E10** and **E13**.

**TABLE 1:** Minimum inhibitory concentration (MIC)^a^ [µg/mL] of triazole derivatives on reference bacterial strains.

Compounds	*E. coli*	*E. coli*	*S. aureus*	*S. aureus*	*S. aureus*	*S. aureus*
	ATCC 25922	DE17>	ATCC 29213	ATCC 43300	ATCC 33731	MRSA2
Vancomycin^b^	—	—	1	2	2	2
Enrofloxacin^c^	0.0625	0.0625	—	—	—	—
E1	>256	>256	128	128	128	>256
E2	>256	>256	128	128	128	>256
E3	64	128	64	64	64	64
E4	128	128	64	64	64	64
E5	128	128	64	64	64	64
E6	128	128	64	64	128	128
E7	>256	>256	128	128	128	128
E8	>256	>256	128	128	128	128
E9	>256	>256	32	32	32	32
E10	32	32	16	16	16	16
E11	>256	>256	256	>256	>256	>256
E12	>256	>256	256	>256	>256	>256
E13	64	64	64	64	64	64
E14	>256	>256	256	256	256	>256
E15	>256	>256	256	256	256	>256
E16	64	128	64	64	64	64
E17	>256	>256	>256	>256	>256	>256
E18	>256	>256	256	>256	256	>256
E19	>256	>256	256	>256	256	>256

^a^ The minimum inhibitory concentration (MIC) is the lowest concentration that completely inhibits microbial growth after 16–24 h. Each experiment was repeated three times.

^b^ Vancomycin is a clinical drug against Gram-positive bacterial.

^c^ Enrofloxacin is a broad-spectrum quinolone-based antibiotic.

#### 2.2.2 Time-killing curve determinations

**FIGURE 2:**
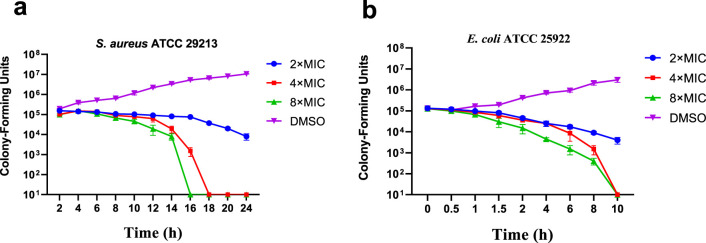
Time-kill kinetics of **E10** against **(A)** *Staphylococcus aureus* ATCC 29213 and **(B)** *Escherichia coli* ATCC 25922. Data are presented as means ± SEM (Standard Error of Mean) from three independent experiments.

In order to evaluate the bactericidal ability of **E10** against *S. aureus* ATCC 29213 and *E. coli* ATCC 25922, we determined the time-kill curves of the compounds by counting the bacterial colonies at different time points using DMSO as a negative control. The results are shown in Figure 2, and the growth of *S. aureus* ATCC 29213 and *E. coli* ATCC 25922 can be completely inhibited at 4-fold MIC.

#### 2.2.3 Drug resistance study

**FIGURE 3:**
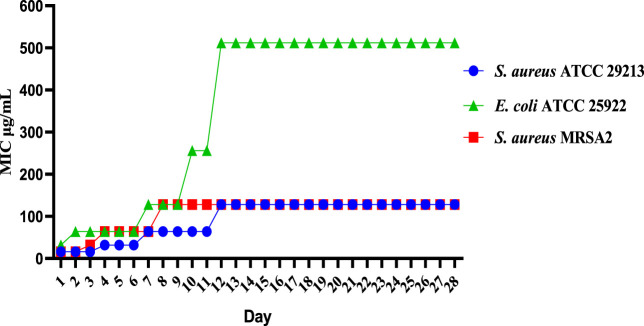
Resistance development of **E10**. Data are presented as means ± SEM from three independent experiments.

The results of the resistance study showed that E10 had a low frequency of spontaneous resistance to *E. coli* ATCC 25922, *S. aureus* ATCC 29213 and MRSA2. As shown in Figure 3, *E. coli* ATCC 25922, *S. aureus* ATCC 29213, and MRSA2 did not increase their MIC values more than 8-fold after 28 generations. These results indicate that E10 can effectively kill bacteria and avoid the development of drug resistance.

### 2.3 The toxicity of the compounds

#### 2.3.1 Hemolysis assay

**FIGURE 4:**
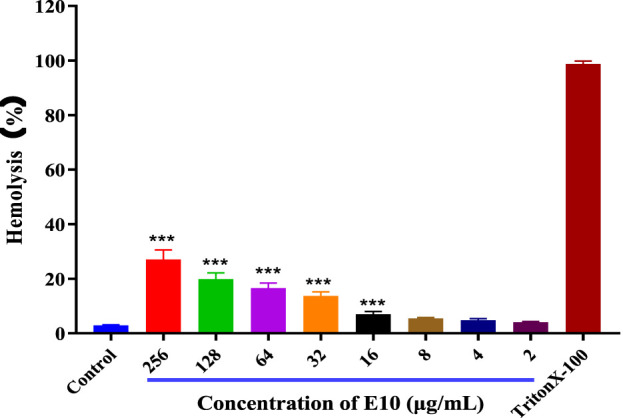
Percentage of hemolysis of rabbit blood cells at various **E10** concentrations. Difference is considered significant at ^*^p < 0.05, ^**^p < 0.01, ^***^p < 0.001. Data are presented as means ± SEM from three independent experiments.

To ensure the safety of our compounds, we first performed a haemolysis test on all compounds. We used 1% Triton X-100 as a positive control and sterile PBS as a negative control compounds in 4% rabbit erythrocytes after incubation at 37°C for 24 h. The results for **E10** are shown in Figure 4. Test compound **E10** at concentrations of 2–256 μg/mL showed no haemolytic properties. This indicates that **E10** does not cause haemolysis of rabbit erythrocytes even at its antibacterial concentration.

#### 2.3.2 Cell cytotoxicity assay

**FIGURE 5:**
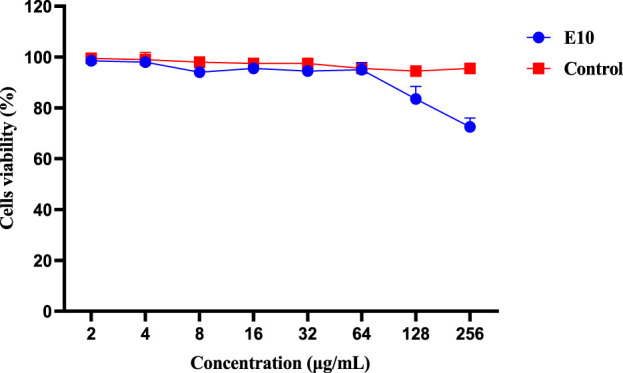
Cytotoxicity of compound **E10** against Vero cells after 24 h. Difference is considered significant at ^*^p < 0.05, ^**^p < 0.01, ^***^p < 0.001. Data are presented as means ± SEM from three independent experiments.

We then evaluated the cytotoxicity of the active compound **E10** against the African green monkey kidney cell line (VERO cells) using the CCK8 assay (Huang et al., 2022). As shown in Figure 5, the maximum inhibitory concentration of **E10** tolerated on VERO cells was 128 μg/mL, indicating that **E10** was not cytotoxic to VERO cells at concentrations up to 64 μg/mL.

### 2.4 Inhibitory effects towards *S. Aureus* biofilm formation

More than 80% of chronic bacterial infections in humans are associated with biofilms, which are surface-associated bacterial communities encased in a secreted exopolysaccharide matrix that resists to environmental and chemical damage (Vishwakarma et al., 2021). Biofilm formation increases bacterial resistance to conventional antibiotic treatments and host immune responses by nearly 1,000-fold (Yang et al., 2024). Failure of antibiotics to eliminate biofilms leads to persistent chronic infections and may promote the development of antibiotic-resistant strains of bacteria. Therefore, there is an urgent need to develop drugs that are effective in preventing biofilm formation and eradicating formed biofilms.

**FIGURE 6:**
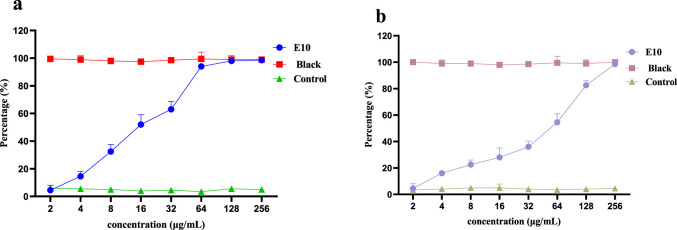
A dose-dependent response was observed in the biofilm inhibition **(A)** and eradication **(B)** activity of compound **E10** against *Staphylococcus aureus* ATCC 29213. Data are presented as means ± SEM from three independent experiments.

Therefore, we investigated whether compound **E10** inhibits biofilm formation in *S. aureus* ATCC 29213. Biofilms were quantified using the crystal violet method and the inhibition of *S. aureus* ATCC 29213 biofilm by compound **E10** at concentrations ranging from 2–256 μg/mL is shown in Figure 6A. **Black** is the group without biofilm formation and **Contol** is the group that formed biofilm without drug. Compound **E10** showed significant biofilm inhibitory activity against *S. aureus* ATCC 29213 with 63.0% inhibition of biofilm at 32 μg/mL.

We next investigated whether compound **E10** could eradicate biofilms formed by *S. aureus* ATCC 29213. **E10** showed significant biofilm eradication activity against *S. aureus* ATCC 29213 by adding **E10** treatment for 24 h after complete growth of biofilm as shown in Figure 6B. Compound **E10** showed 54.5% eradication from biofilm at a concentration of 64 μg/mL.

### 2.5 The anti-inflammatory activity of the compounds

**FIGURE 7:**
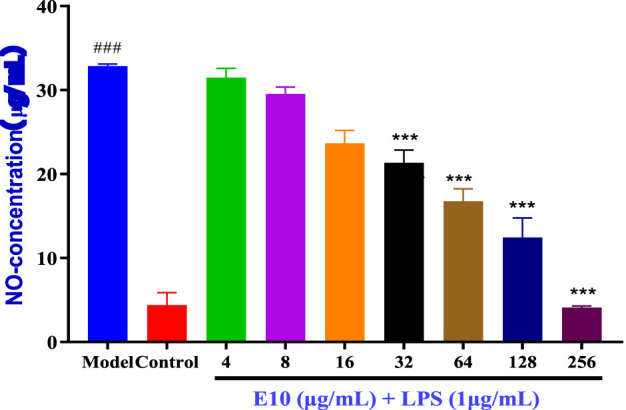
Anti-inflammatory activity of the **E10** compounds in RAW 264.7 macrophage cells was evaluated in the LPS-enhanced leukocyte migration assay. Compared with the LPS model group, ^*^p < 0.05, ^**^p < 0.01, ^***^p < 0.001; ^###^p < 0.001 vs. control group. Data are presented as means ± SEM from three independent experiments.

Inflammation often accompanies the onset of infection, and since triazole derivatives have been reported to inhibit inflammation, we further examined the effect of compound **E10** on the levels of the inflammatory factor NO (Jiaranaikulwanitch et al., 2021). The results are shown in Figure 7, where LPS alone significantly induced NO production in RAW 264.7 cells compared to NO produced by the control, and treatment with the studied compound **E10** affected NO levels. **Model** is the inflammation forming group and **Control** is the normal without inflammation group. Compound **E10** showed significant inhibition of NO production at concentrations 16 μg/mL.

**FIGURE 8:**
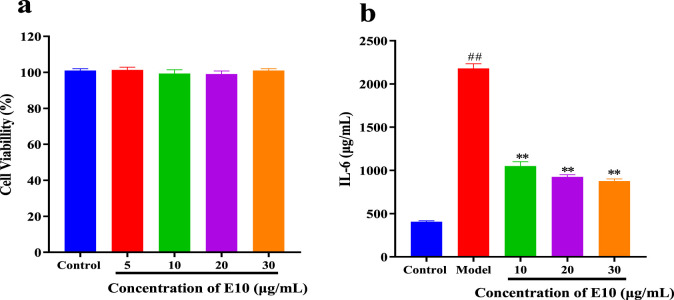
**(A)** Cytotoxic activity of **E10** against BEAS-2B cells. **(B)** Level of IL-6 treated with **E10** in IL-4/TNF-α-stimulated BEAS-2B cells. ^*^p < 0.05, ^**^p < 0.01, ^***^p < 0.001; ^###^p < 0.001 vs. control group. Data are presented as means ± SEM from three independent experiments.

To further expand the drug application scenario, we performed other inflammatory factor assays. We know that allergic asthma is the airway disease caused by some abnormal inflammatory factors. Th2 cells play an important role in triggering and pushing airway inflammation by producing, e.g., interleukin (IL)-4, (IL)-6 and TNF-α, cytokines that exacerbate the disease with immunoglobulin E (IgE) and mucus production (Li et al., 2022). We preliminarily evaluated the cytotoxicity of **E10** using the CCK8 assay, and as shown in Figure 8A, **E10** showed no significant cytotoxic effects on BEAS-2B cells at concentrations below 30 μg/mL. Next, we investigated the anti-inflammatory effect of **E10** on IL-4/TNF-α- stimulated BEAS-2B cells. As shown in Figure 8B, **E10** inhibited the IL-4/TNF-α-induced production of the pro-inflammatory cytokine IL-6. This suggests that **E10** deserves further exploration in the treatment of asthma.

### 2.6 Antimicrobial mechanism investigation

#### 2.6.1 Membrane depolarization and permeabilization assay

It has been reported that the antibacterial activity of Schiff bases is related to their hydrophobicity, which favours membrane interactions with pathogenic bacteria (Pervaiz et al., 2022). Based on the action of compound **E10** on bacterial cell membranes, it may cause changes in bacterial cell membrane depolarisation and permeability. We therefore investigated the effect of **E10** on the depolarisation and permeability of bacterial membranes using the fluorescent probes 3,3-dipropylthiodicyanoiodide (DISC35) and SYTOX Green (Yang et al., 2022; Kong et al., 2023).

**FIGURE 9:**
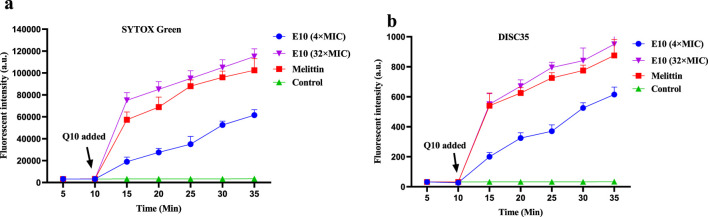
Antibacterial mechanism of **E10** against *Staphylococcus aureus* ATCC 29213. **(A)** Cytoplasmic membrane permeabilization ability of **E10** using a SYTOX Green assay. **(B)** Cytoplasmic membrane depolarization of **E10** using the DiSC35 probe. The blank control was bacteria without compound treatment, and melittin was used as a reference drug. Data are presented as means ± SEM from three independent experiments.

After 10 min, compound **E10** was added to a solution of *S. aureus* ATCC 29213 containing the fluorescent probes DiSC35 or SYTOX Green, and the fluorescence intensity of the mixed bacterial solution continued to increase (Figures 9A, B). When the **E10** concentration was 4×MIC or 32×MIC, the fluorescence intensity of the mixed bacterial solution at 35 min was significantly increased compared to the initial fluorescence intensity, while the fluorescence intensity of the blank control without **E10** remained unchanged. This suggests that **E10** perturbs the positive and negative polarisation states of the interior and exterior of the bacterial cell membrane, thereby enhancing permeability. Similarly, the fluorescence intensity of melittin, a membrane-targeted natural antimicrobial peptide, was significantly enhanced in the positive control group. These results suggest that **E10** can affect the bacterial cell membrane, leading to bacterial death.

#### 2.6.2 Leakage of proteins and DNA

**FIGURE 10:**
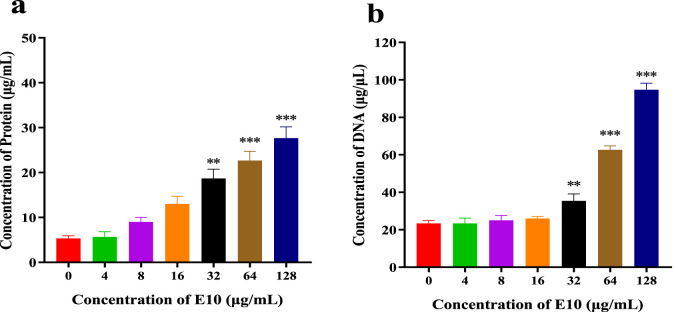
**(A)** DNA leakage resulting from the treatment of **E10** on *Staphylococcus aureus* ATCC 29213. **(B)** Protein leakage caused by the treatment of **E10** on *Staphylococcus aureus* ATCC 29213. ^*^p < 0.05, ^**^p < 0.01, ^***^p < 0.001. Data are presented as means ± SEM from three independent experiments.

We further determined the changes in intracellular protein and DNA concentrations after the addition of different concentrations of **E10** to *S. aureus* ATCC 29213 medium (Raman et al., 2014). Compared with the blank control group, **E10** treatment of *S. aureus* ATCC 29213 resulted in a significant and dose-dependent increase in the concentration of leaked proteins and DNA (Figures 10A, B). This suggests that **E10** can cause membrane damage in *S. aureus* ATCC 29213 cells, leading to leakage of intracellular proteins and DNA.

### 2.7 Screening of heavy metals and metal ion removal from aqueous solution

**FIGURE 11:**
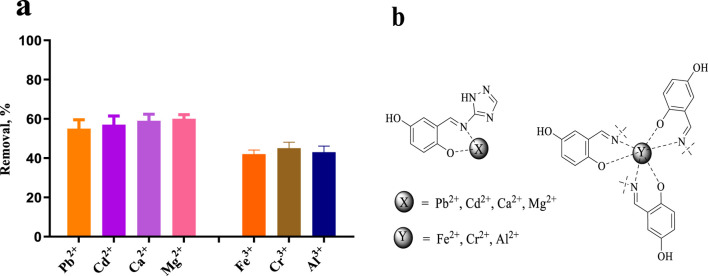
**(A)** Removal efficiencies of Pb^2+^, Cd^2+^, Ca^2+^, Mg^2+^, Fe^3+^, Cr^3+^ and Al^3+^ using compound **E10**, **(B)** Possible mechanism of metal and heavy metal ion removal. Data are presented as means ± SEM from three independent experiments.

We previously speculated that **E10** should have good metal chelating ability to remove heavy metal ions from water, or be modified to act as a molecular probe to detect water quality. pH is a key parameter for the adsorption of heavy metals and metal ions. Since metal ions may interact with hydroxyl ions in water when the pH value is greater than 7, affecting the accuracy of our determination, the adsorption experiments in this study were carried out at a pH value of 6.0. After testing, we found that, as shown in Figure 11A, the removal amount of **E10** for divalent metal ions is 55%–60%, and the removal rate of **E10** for trivalent metal ions is 42%–45%. We infer that the removal rate of metal ions is related to the coordination form of the compound to the metal ion. One possible mechanism is shown in Figure 11B, where **E10** chelates 1:1 with divalent metal ions and 3:1 with trivalent metal ions, which also directly reduces its removal of trivalent metals.

## 3 Conclusion

In this paper, 3-amino-1,2,4-triazole (**D**) was synthesised using thiourea as a starting material, and finally the coupling end product **E** of triazole and Schiff base was obtained by aldolamine condensation reaction, and the structures of all the compounds were determined by spectroscopic analysis. *In vitro* antimicrobial activity showed that **E10** had a MIC of 32 μg/mL against the tested *E. coli* and 16 μg/mL against the tested *S. aureus* strains. Meanwhile, **E10** has a good anti-biofilm effect. Antibacterial mechanism studies have shown that **E10** has a good membrane targeting ability, thus disrupting cell membranes, leading to leakage of intracellular proteins and DNA and accelerating bacterial death. In terms of anti-inflammation, **E10** dose-dependently inhibits the levels of inflammatory factors NO and IL-6, which deserves further exploration in the treatment of asthma. The study of metal ion removal capacity showed that the synthesised triazole derivatives have high capacity to remove heavy metals Pb^2+^, Cd^2+^, Ca^2+^, Mg^2+^, Fe^3+^, Cr^3+^ and Al^3+^ in the range of 42%–60%. Next step, we will use **E10** as a lead compound to further optimise its antimicrobial capacity and investigate its antimicrobial targets in depth.

## 4 Experimental section

### 4.1 Chemically synthetical experiments

#### 4.1.1 Methyl-isothiourea (B)

We added iodomethane solution (1.7 g, 12 mmol) dropwise to an ethanol (10 mL) solution of thiourea (0.76 g, 10 mmol) at room temperature. The mixture was stirred at room temperature for 12 h and the solvent was spun off at the end of the reaction. Final recrystallisation from methanol gave white solid **B** (2.2 g, yield: 93%). ^1^H NMR (400 MHz, dmso) δ 7.76 (s, 1H), 7.21 (s, 1H), 2.69 (s, 3H). TOF-MS, m/z: [M + H^+^], calcd. for C_2_H_7_N_2_S^+^, 91.0330, found: 91.0337.

#### 4.1.2 Hydrazinecarboximidamide (C)

**B** (1 mmol) was dissolved in ethanol, appropriate amount of sodium hydroxide was added to adjust the pH to neutral and stirred for 10 min, then hydrazine hydrate (2 mmol) was added and the reaction was heated to 50°C for 6 h. The solvent was spun off, and the target product **C** (66 mg, yield: 88%) was finally recrystallised from ethanol. ^1^H NMR (400 MHz, dmso) δ 8.82 (s, 1H), 4.67 (s, 2H). TOF-MS, m/z: [M + H^+^], calcd. for CH_7_N_4_^+^, 75.0670, found: 75.0675.

#### 4.1.3 1H-1,2,4-triazol-5-amine (D)

We added sodium hydroxide 7.5 mmol, 5 mL of water to aminoguanidine **C** (7.5 mmol) and stirred the reaction for 10 min. Then formic acid (7.40 mmol) was added, and the reaction mixture was heated to 80°C to dissolve all of it, and then was dissolved at 120°C for 6 h. The reaction was stopped by the addition of ice water. After the reaction was stopped, the reaction was stopped by adding ice water and recrystallised from ethanol to give a white solid **D** (541 mg, yield: 85%) (Manning et al., 2002; Stojković et al., 2023). ^1^H NMR (400 MHz, dmso) δ 7.43 (s, 1H), 5.75 (s, 2H). TOF-MS, m/z: [M + H^+^], calcd. for C_2_H_5_N_4_^+^, 85.0514, found: 85.0519.

#### 4.1.4 (E)-1-(2-chloropyridin-4-yl)-N-(1H-1,2,4-triazol-5-yl)methanimine (E1)

We dissolved the product **D** (1 mmol) in anhydrous ethanol, added aldehyde derivatives with different substituents, such as 2-chloroisonicotinaldehyde, and reacted at reflux for 8 h. After the reaction was stopped, the reaction solution was concentrated and finally the end product **E1** (104 mg, yield: 50%) was obtained by recrystallisation from ethanol. The rest of the E-series products were obtained in the same way as **E1** and will not be repeated thereafter. ^1^H NMR (400 MHz, DMSO-*d*_6_) δ 9.23 (s, 1H), 8.59 (s, 2H), 7.97 (d, 2H). ^13^C NMR (100 MHz, DMSO) δ 160.16, 156.93, 148.96, 147.02, 125.31, 121.88. TOF-MS, m/z: [M + H^+^], calcd. for C_8_H_7_ClN_5_^+^, 208.0390, found: 208.0395.

#### 4.1.5 (E)-1-(4-(methylsulfonyl)phenyl)-N-(1H-1,2,4-triazol-5-yl) methanimine (E2)

Compound **E2** (205 mg, Yield: 82%). ^1^H NMR (400 MHz, DMSO-*d*_6_) δ 9.31 (s, 1H), 8.24 (d, *J* = 8.1 Hz, 2H), 8.13 (s, 1H), 8.06 (d, *J* = 8.1 Hz, 2H), 7.63 (s, 1H), 3.27 (s, 3H). ^13^C NMR (101 MHz, DMSO-d6) δ 193.33, 193.12, 145.80, 139.78, 130.69, 130.22, 128.22, 128.01, 43.78. TOF-MS, m/z: [M + H^+^], calcd. for C_10_H_11_N_4_O_2_S^+^, 251.0602, found: 251.0609.

#### 4.1.6 (E)-2-(((1H-1,2,4-triazol-5-yl)imino)methyl)-5-methylphenol (E3)

Compound **E3** (162 mg, Yield: 80%). ^1^H NMR (400 MHz, DMSO-*d*_6_) δ 9.34 (s, 1H), 8.55 (s, 1H), 7.55 (s, 1H), 7.24 (d, *J* = 7.9 Hz, 1H), 6.87 (d, *J* = 8.4 Hz, 1H), 2.25 (s, 3H). ^13^C NMR (100 MHz, DMSO) δ 158.68, 144.74, 137.69, 132.59, 129.32, 128.54, 122.35, 117.62, 117.03, 20.37. TOF-MS, m/z: [M + H^+^], calcd. for C_10_H_11_N_4_O^+^, 203.0933, found: 203.0940.

#### 4.1.7 (E)-1-(quinolin-4-yl)-N-(1H-1,2,4-triazol-5-yl)methanimine (E4)

Compound **E4** (188 mg, Yield: 85%). ^1^H NMR (400 MHz, DMSO-d6) δ 9.81 (s, 1H), 9.06 (d, *J* = 8.8 Hz, 1H), 8.63 (s, 0H), 8.11 (dd, *J* = 18.4, 6.3 Hz, 1H), 7.80 (dt, *J* = 37.7, 7.2 Hz, 1H), 4.33 (s, 1H), 3.42 (q, *J* = 6.9 Hz, 1H), 1.03 (td, *J* = 7.0, 1.1 Hz, 2H). ^13^C NMR (101 MHz, DMSO-*d*_6_) δ 151.14, 149.01, 144.82, 129.60, 128.58, 126.29, 125.05, 124.71, 123.80, 123.21. TOF-MS, m/z: [M + H^+^], calcd. for C_12_H_10_N_5_^+^, 224.0936, found: 224.0946.

#### 4.1.8 (E)-1-(3,5-dibromopyridin-4-yl)-N-(1H-1,2,4-triazol-5-yl)methanimine (E5)

Compound **E5** (183 mg, Yield: 55%). ^1^H NMR (400 MHz, DMSO-*d*_6_) δ 10.09 (s, 0H), 9.22 (s, 1H), 8.98–8.78 (m, 3H). ^13^C NMR (100 MHz, DMSO) δ 161.07, 152.25, 151.64, 144.95, 120.95, 119.95. TOF-MS, m/z: [M + H^+^], calcd. for C_8_H_6_BrN_5_^+^, 331.8969, found: 331.8975.

#### 4.1.9 (E)-1-(2-bromopyridin-4-yl)-N-(1H-1,2,4-triazol-5-yl)methanimine (E6)

Compound **E6** (192 mg, Yield: 58%). ^1^H NMR (400 MHz, DMSO-d6) δ 9.23 (s, 1H), 8.59 (d, *J* = 16.6 Hz, 2H), 8.16 (s, 1H), 7.99 (s, 1H). ^13^C NMR (100 MHz, DMSO) δ 160.53, 159.10, 153.65, 147.52, 146.81, 132.95, 128.57, 125.34. TOF-MS, m/z: [M + H^+^], calcd. for C_8_H_7_BrN_5_^+^, 251.9885, found: 251.9889.

#### 4.1.10 (E)-1-([1,1′-biphenyl]-4-yl)-N-(1H-1,2,4-triazol-5-yl)methanimine (E7)

Compound **E7** (226 mg, Yield: 91%). ^1^H NMR (400 MHz, DMSO-d6) δ 9.26 (d, *J* = 7.6 Hz, 1H), 8.53 (s, 1H), 8.08 (t, *J* = 8.4 Hz, 2H), 7.96–7.65 (m, 4H), 7.61–7.29 (m, 3H). ^13^C NMR (101 MHz, DMSO) δ 166.69, 165.53, 163.18, 144.42, 143.97, 139.65, 135.05, 130.64, 130.13, 129.56, 128.65, 127.76, 127.63, 127.36. TOF-MS, m/z: [M + H^+^], calcd. for C_15_H_13_N_4_^+^, 249.1140, found: 249.1146.

#### 4.1.11 (E)-1-(4-(tert-butyl)phenyl)-N-(1H-1,2,4-triazol-5-yl)methanimine (E8)

Compound **E8** (201 mg, Yield: 88%). ^1^H NMR (400 MHz, DMSO-*d*_6_) δ 9.19 (s, 1H), 7.93 (d, *J* = 8.0 Hz, 2H), 7.57 (d, *J* = 8.1 Hz, 2H), 1.32 (s, 9H). ^13^C NMR (101 MHz, DMSO-*d*_6_) δ 164.20, 155.96, 133.16, 129.91, 129.57, 126.32, 35.34, 31.35. TOF-MS, m/z: [M + H^+^], calcd. for C_13_H_17_N_4_^+^, 229.3068, found: 229.3075.

#### 4.1.12 (E)-2-(((1H-1,2,4-triazol-5-yl)imino)methyl)-5-nitrophenol (E9)

Compound **E9** (138 mg, Yield: 62%). ^1^H NMR (400 MHz, DMSO-d6) δ 9.49 (s, 1H), 8.76 (s, 1H), 8.23 (d, *J* = 12.0 Hz, 1H), 7.11 (d, *J* = 9.1 Hz, 1H). ^13^C NMR (100 MHz, DMSO) δ 165.71, 162.05, 140.29, 131.07, 129.23, 126.84, 124.82, 120.25, 118.24. TOF-MS, m/z: [M + H^+^], calcd. for C_9_H_8_N_5_O_3_^+^, 224.0627, found: 224.0634.

#### 4.1.13 (E)-2-(((1H-1,2,4-triazol-5-yl)imino)methyl)benzene-1,4-diol (E10)

Compound **E10** (139 mg, Yield: 68%). ^1^H NMR (400 MHz, DMSO-d6) δ 11.63 (s, 1H), 9.32 (d, *J* = 20.1 Hz, 1H), 9.09 (s, 1H), 8.54 (s, 1H), 7.13 (s, 1H), 6.99–6.73 (m, 2H). ^13^C NMR (100 MHz, DMSO) δ 164.69, 153.69, 150.31, 144.68, 124.99, 122.38, 119.75, 117.83, 116.77. TOF-MS, m/z: [M + H^+^], calcd. for C_9_H_9_N_4_O_2_^+^, 205.0725, found: 205.0725.

#### 4.1.14 (E)-4-(((1H-1,2,4-triazol-5-yl)imino)methyl)-2,6-dimethylphenol (E11)

Compound **E11** (168 mg, Yield: 78%). ^1^H NMR (400 MHz, DMSO-d6) δ 9.01 (s, 1H), 7.58 (s, 2H), 2.23 (s, 6H). ^13^C NMR (100 MHz, DMSO) δ 158.26, 151.29, 148.40, 145.02, 130.34, 130.30, 125.15, 17.04. TOF-MS, m/z: [M + H^+^], calcd. for C_11_H_13_N_4_O^+^, 217.1089, found: 217.1095.

#### 4.1.15 (E)-1-(4-chlorophenyl)-N-(1H-1,2,4-triazol-5-yl)methanimine (E12)

Compound **E12** (165 mg, Yield: 80%). ^1^H NMR (400 MHz, DMSO-d6) δ 9.21 (s, 1H), 8.01 (d, *J* = 7.9 Hz, 2H), 7.59 (d, *J* = 7.2 Hz, 2H). ^13^C NMR (100 MHz, DMSO) δ 160.74, 158.57, 149.77, 136.42, 133.98, 130.60, 128.96. TOF-MS, m/z: [M + H^+^], calcd. for C_9_H_8_ClN_4_^+^, 207.0617, found: 207.0625.

#### 4.1.16 (E)-2-(((1H-1,2,4-triazol-5-yl)imino)methyl)-4-chlorophenol (E13)

Compound **E13** (138 mg, Yield: 62%). ^1^H NMR (400 MHz, DMSO-d6) δ 10.20 (s, 0H), 9.39 (s, 1H), 7.87 (s, 1H), 7.43 (dd, *J* = 8.8, 2.6 Hz, 1H), 7.00 (d, *J* = 8.8 Hz, 1H). ^13^C NMR (100 MHz, DMSO) δ 190.18, 163.28, 159.97, 159.31, 136.18, 127.83, 123.94, 119.99, 119.20. TOF-MS, m/z: [M + H^+^], calcd. for C_9_H_8_ClN_4_O^+^, 223.0386, found: 223.0394.

#### 4.1.17 (E)-1-(pyridin-4-yl)-N-(1H-1,2,4-triazol-5-yl)methanimine (E14)

Compound **E14** (121 mg, Yield: 70%). ^1^H NMR (400 MHz, DMSO-d6) δ 9.24 (s, 1H), 8.87 (s, 1H), 8.76 (d, *J* = 5.3 Hz, 2H), 7.96–7.86 (m, 3H), 7.79 (d, *J* = 5.1 Hz, 1H). ^13^C NMR (100 MHz, DMSO) δ 160.68, 158.26, 149.78, 146.71, 143.30, 124.53. TOF-MS, m/z: [M + H^+^], calcd. for C_8_H_8_N_5_^+^, 174.0779, found: 174.0785.

#### 4.1.18 (E)-1-p-tolyl-N-(1H-1,2,4-triazol-5-yl)methanimine (E15)

Compound **E15** (166 mg, Yield: 89%). ^1^H NMR (400 MHz, DMSO-d6) δ 9.95 (s, 1H), 7.80 (d, *J* = 11.2 Hz, 2H), 7.42 (d, *J* = 10.4 Hz, 2H), 2.40 (s, 3H). ^13^C NMR (100 MHz, DMSO) δ 160.68, 157.46, 147.23, 142.67, 130.55, 129.45, 126.36, 23.24. TOF-MS, m/z: [M + H^+^], calcd. for C_10_H_11_N_4_^+^, 187.0983, found: 187.0988.

#### 4.1.19 (E)-1-(4-nitrophenyl)-N-(1H-1,2,4-triazol-5-yl)methanimine (E16)

Compound **E16** (195 mg, Yield: 90%). ^1^H NMR (400 MHz, DMSO-d6) δ 9.33 (s, 1H), 8.45–8.15 (m, 4H). ^13^C NMR (100 MHz, DMSO) δ 160.53, 158.93, 149.77, 146.86, 141.38, 128.96, 124.51. TOF-MS, m/z: [M + H^+^], calcd. for C_9_H_8_N_5_O_2_^+^, 218.0678, found: 218.0685.

#### 4.1.20 (E)-1-(4-methoxyphenyl)-N-(1H-1,2,4-triazol-5-yl)methanimine (E17)

Compound **E17** (141 mg, Yield: 70%). ^1^H NMR (400 MHz, DMSO-d6) δ 9.12 (s, 1H), 8.48 (s, 1H), 7.93 (d, *J* = 7.4 Hz, 2H), 7.06 (d, *J* = 7.9 Hz, 2H), 3.82 (s, 3H). ^13^C NMR (100 MHz, DMSO) δ 164.68, 162.92, 151.32, 144.20, 132.26, 131.35, 130.11, 114.95, 56.12. TOF-MS, m/z: [M + H^+^], calcd. for C_10_H_11_N_4_O^+^, 203.0933, found: 203.0937.

#### 4.1.21 (E)-4-(((1H-1,2,4-triazol-5-yl)imino)methyl)phenol (E18)

Compound **E18** (160 mg, Yield: 85%). ^1^H NMR (400 MHz, DMSO-d6) δ 10.31 (s, 1H), 9.76 (s, 0H), 9.06 (s, 1H), 7.82 (d, *J* = 8.4 Hz, 2H), 6.89 (d, *J* = 8.3 Hz, 2H). ^13^C NMR (100 MHz, DMSO) δ 163.79, 161.81, 137.04, 135.11, 132.57, 129.15, 116.31. TOF-MS, m/z: [M + H^+^], calcd. for C_9_H_9_N_4_O^+^, 189.0776, found: 189.0779.

#### 4.1.22 (E)-4-(((1H-1,2,4-triazol-5-yl)imino)methyl)benzoic acid (E19)

Compound **E19** (173 mg, Yield: 80%). ^1^H NMR (400 MHz, DMSO-d6) δ 10.31 (s, 1H), 9.76 (s, 0H), 9.06 (s, 1H), 7.82 (d, *J* = 8.4 Hz, 2H), 6.89 (d, *J* = 8.3 Hz, 2H). ^13^C NMR (100 MHz, DMSO) δ 170.05, 160.08, 158.65, 149.17, 141.01, 130.30, 128.74, 124.04. TOF-MS, m/z: [M + H^+^], calcd. for C_10_H_9_N_4_O_2_^+^, 217.0725, found: 217.0733.

### 4.2 The antibacterial activity of the compounds

#### 4.2.1 Determination of minimum inhibitory concentration

Minimum Inhibitory Concentrations (MIC) were determined for all compounds according to Clinical and Laboratory Standards Institute (CLSI) guidelines (Mei et al., 2023). Detailed procedures can be found in the Supplementary Information.

#### 4.2.2 Time-killing kinetics

We determined time-kill kinetics by plate colony counting. Details of the operation can be found in the previously reported literature (Song et al., 2021).

#### 4.2.3 Drug resistance study

The drug resistance study is similar to the MIC assay, but the process needs to be repeated for 28 days, and some details can be found in the literature (Yang et al., 2024).

### 4.3 The toxicity of the compounds

#### 4.3.1 Hemolysis assay

The detailed procedure for the hemolysis experiment is included in the Supplementary Information (Xu et al., 2024).

#### 4.3.2 Cytotoxicity assay

The cytotoxicity test was conducted according to previous literature reports (Xu et al., 2024). Cell viability was assessed using the cell counting kit 8 (Beyotime, Shanghai, China) method. Cytotoxicity assay was done as described before with minor modifications. The results were calculated as follows: Cell viability (%) = (OD_450_ sample value − OD_450_ blank hole value)/(OD_450_ value of untreated control − OD_450_ blank hole value) × 100%.

### 4.4 Biofilm formation assay

Detailed procedures for biofilms are provided in the Supplementary Information (Etayash et al., 2021).

### 4.5 The anti-inflammatory activity of the compounds

LPS-stimulated RAW 264.7 cells were studied using methods reported in the literature (Jiaranaikulwanitch et al., 2021).

#### 4.5.1 BEAS-2B Cell culture and viability. Human bronchial epithelial

Detailed procedures are provided in the Supplementary Information (Li et al., 2022).

#### 4.5.2 Anti-inflammatory assay in BEAS-2B Cells

Detailed procedures are provided in the Supplementary Information (Li et al., 2022).

### 4.6 Membrane depolarization study

Detailed procedures for biofilms are provided in the Supplementary Information (Yang et al., 2024).

### 4.7 DNA and protein leakage

We refer to the literature for our experiments (Lu et al., 2023). Detailed procedures for biofilms are provided in the Supplementary Information.

### 4.8 Adsorption studies

Determination of metal ion removal was carried out with reference to previously reported methods (Xu et al., 2024; Hozien et al., 2020). We only determined a few more trivalent metal ions using the previous method. The removal percentage (Removal, %), was calculated as presented in Equation: Removal, % = [(C_0_-C_e_)/C_0_] × 100.

### 4.9 Statistical analysis

The above experimental data is the average ±SEM independent experiment of three data points. One-way analysis of variance was used to process the statistical differences between the two groups.

## Data Availability

The original contributions presented in the study are included in the article/Supplementary Material, further inquiries can be directed to the corresponding author.
