# Supplementary material for: Discovery of triazole derivatives for biofilm disruption, anti-inflammation and metal ion chelation
Source: Front Chem. 2025 Feb 26;13:1545259. doi: 10.3389/fchem.2025.1545259 (PMC11897050; doi:10.3389/fchem.2025.1545259)
Supplement: Supplementary file 1 [file DataSheet1.doc]

**Discovery of triazole derivatives for biofilm disruption, anti-inflammation and metal ion chelation**

Shuang Hong^1^, Hongzhi Lu^1^*[[1]](#footnote-2), Dawei Tian^1^, Yue Chang^1^, Qi Lu^1^, Feng Gao^1^

*^1^Department of Pediatrics, Maternity and Child Health Hospital of Qinhuangdao, Qinhuangdao, China, 066000, Qinhuangdao, China*

**Table of Contents**

**Determination of Minimum Inhibitory Concentration**···································1

**Hemolysis assay**·····························································································1

**Biofilm Formation Assay**···············································································1

**The anti-inflammatory activity of the compounds**··········································2

**BEAS-2B Cell Culture and Viability**······························································2

**Anti-Inflammatory Assay in BEAS-2B Cells**··················································3

**Membrane depolarization study**····································································3

**DNA and protein leakage**··············································································4

**Spectral data**······························································································5-25

**Determination of Minimum Inhibitory Concentration**

The minimum inhibitory concentrations (MICs) of all the test compounds were determined using microdilution method according to the Clinical and Laboratory Standards Institute (CLSI) guidelines (Mei et al., 2023). The bacterial strains were added to Mueller−Hinton broth (MHB) and cultured on a shaker for approximately 5 h at 37°C, then diluted to a final concentration of 10^5^ cfu/mL in a 96-well microtiter plate. The compounds were then added at a series concentration (0.05, 0.1, 0.25, 0.5, 1, 2, 4, 8, 16, 32, 64, 128, 256 μg/mL) and the plate was incubated at 37°C for 18 h. The MICs are determined as the minimum concentration of the visually clear wells. Three independent assays were performed for all the tests.

**Hemolysis assay**

The hemolysis assay was conducted following a previously reported procedure (Xu et al., 2024). One hundred microliters of **E10** at different concentrations in PBS (Phosphate-Buffered Saline) was mixed with 100 µL PBS containing 4% defibrinated rabbit erythrocytes, resulting in a final concentration of 4, 8, or 16 µg/mL, respectively. We used 1% Triton X-100 as a positive control and sterile PBS as a negative control. After incubation at 37°C for 1 h, the erythrocytes were isolated from the supernatant by centrifugation at 1000 g for 5 min. Subsequently, the absorbance at 490 nm was recorded. The hemolysis ratio was calculated using the following formula: hemolysis (%) = (sample − PBS) / (Triton − PBS) × 100%. The experiments were repeated thrice.

**Biofilm Formation Assay**

Biofilms were quantified using the crystal violet method (Etayash et al., 2021). The *S. aureus* ATCC 29213 was then diluted 100-fold with fresh TSB medium containing 1% w/v glucose, and the diluted bacterial and compound solutions were added to 96-well plates containing TSB (200 μL) containing 1% w/v glucose. **E10** was added directly to the wells at concentrations ranging from 256 to 2 μg/ml to assess the concentration that inhibited biofilm formation. The same amount of DMSO was added to each well in the control group. the bacterial cultures were removed, rinsed three times with PBS and air dried; then 0.1% crystal violet solution was added to each well and stained for 15 minutes. After removing the excess crystal violet solution and rinsing three times with PBS, the pigment was dissolved in 95% ethanol. Finally, absorbance values were measured at 595 nm using a microplate reader. The biofilm inhibition was calculated as OD_595_ control − OD_595_/OD_595_ control × 100%.

In this test, the bacterial biofilm was formed prior to the addition of the treatment compounds. First, a suspension of bacteria in TSB with 1 % v/v glucose added was added to each well of a 96-well microtiter plate and incubated at 37°C for 24 hours. Then, sub-microgram values of compound Q10 was added directly to the wells at concentrations ranging from 256 to 2 µg/ml to assess the concentration that eliminated the percentage of biofilm formation. Other steps were similar to the inhibition experiments.

**The anti-inflammatory activity of the compounds**

LPS-stimulated RAW 264.7 cells were studied using methods reported in the literature (Jiaranaikulwanitch et al., 2021). All cells were treated for 24 h with the compounds studied and LPS or LPS alone. To determine the level of NO production, nitrite accumulation was used as an indicator of NO production using a microtiter plate method based on the Griess reaction. The NO level of each of the tested cell supernatants was expressed as NO level of the tested cell supernatant ×100/NO level of the control.

**BEAS-2B Cell Culture and Viability**

BEAS-2B cells were grown and maintained in Dulbecco’s modified Eagle medium (DMEM, Thermo Fisher Scientific, Shanghai, China) in a 5% CO2 humidified environment at 37°C. The cells (1 × 10^5^/well) were seeded in a 96-well microplate and cultured for 24 h, and then treated with the sample for 24 h. Then, each well was treated with CCK8 solution (10 μL) and incubated at 37 °C for 2 h. The cell viability was determined at 450nm according to the product instruction manual (Promega).

**Anti-Inflammatory Assay in BEAS-2B Cells**

The anti-inflammatory effects of all fractions and components against airway inflammation were assayed using an IL-4/TNF-α-induced BEAS-2B cell model.20,21 The cells were inoculated in 24-well plates (500 μL/well, 1 × 10^5^ cells/mL) for 24 h, the test sample was added and mixed for 60 min, and then IL-4 (10 ng/mL) and TNF-α (10 ng/mL) were added and incubated for 24 h. According to the manufacturer’s protocol, the production of IL-6 was detected based on an ELISA kit.

All experiments were performed in triplicate.

**Membrane depolarization study**

We operated with reference to the methods reported in the literature (Yang et al., 2024). A single *S. aureus* ATCC 29213 colony was picked and added to LB broth and incubated on a shaker for 6 hours. The supernatant was then removed by centrifugation for 5 min. It was washed three times with PBS buffer and finally resuspended with an equal amount of PBS. The suspension (1 × 10^8^ CFU/mL, 150 μL) was added to a black 96-well plate, then DiSC35 (10 μM, 40 μL) was added in the dark and the mixture was incubated for 30 min at 37°C in the dark. Fluorescence intensity was measured continuously at 5-minute intervals for 40 min at an excitation wavelength of 622 nm and an emission wavelength of 670 nm, and then **E10** solution (10 μL) was added. The fluorescence intensity was measured every 5 minutes for 40 minutes. Sterile water was used as a negative control and melittin (32 μg/ mL) as a positive control, and the experiment was repeated three times.

We operated with reference to the methods reported in the literature (Yang et al., 2024). *S. aureus* ATCC 29213 suspension (10^8^ CFU/mL, 150 μL) was added to a black 96-well plate, followed by SYTOX Green solution (3 μM, 40 μL) in the dark, and incubated at 37°C for 30 min. The excitation wavelength is 500 nm, the emission wavelength is 530 nm, and the fluorescence intensity is measured every 5 minutes for 40 minutes. **E10** isodilution (10 μL) was then added to give final concentrations of 64 and 256 μg/mL, respectively, and fluorescence intensity was measured at 5-minute intervals over a 40-minute period.Sterile water was used as a negative control and melittin (32 μg/ mL) as a positive control, and the experiment was repeated three times.

**DNA and protein leakage**

We refer to the literature for our experiments (Lu et al., 2023). Different concentrations of **E10** solution were added to *S. aureus* ATCC 29213 solution (2 × 10^6^ CFU/mL) at final concentrations of 8 × MIC, 4 × MIC, 2 × MIC, 1 × MIC, 1/2 × MIC and 1/4 × MIC, respectively. It was then incubated at37°C for 4 h, centrifuged, and the supernatant was aspirated for determination. DNA concentration was determined using a microspectrophotometer; protein concentration was determined using the BCA Protein Concentration Assay Kit.


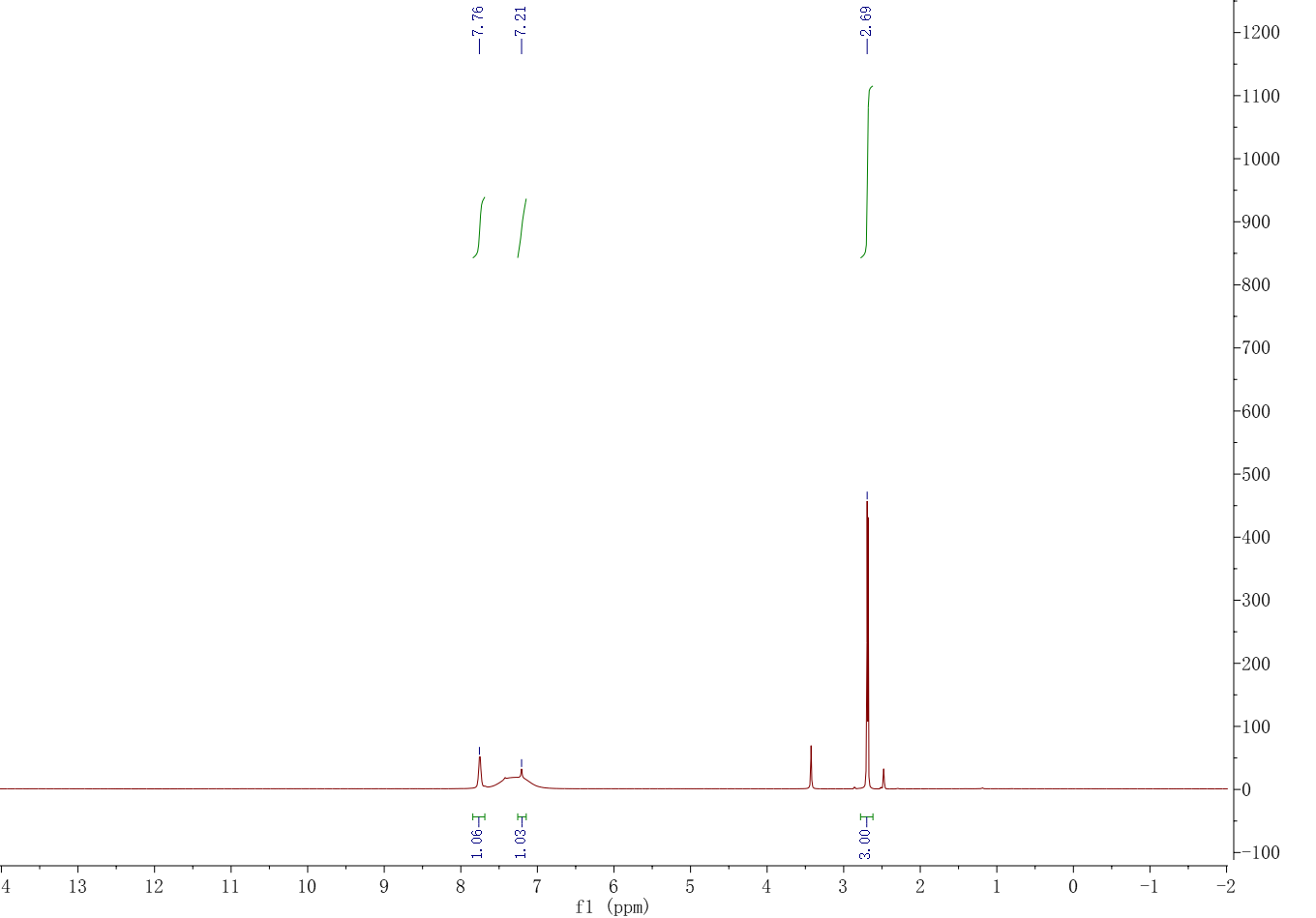


Fig. *^1^H NMR of* **B** (400 MHz, DMSO)


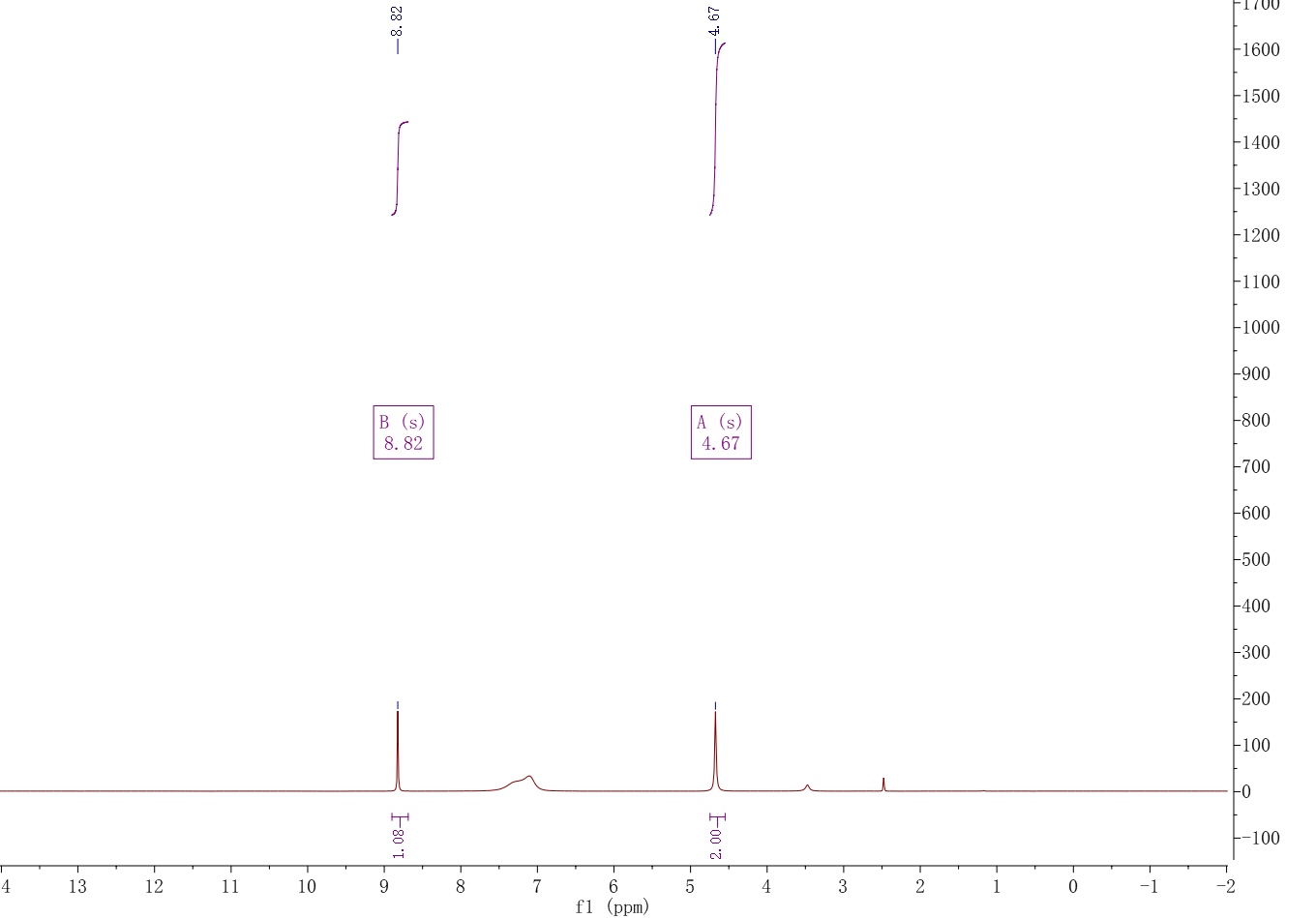


Fig. *^1^H NMR of* **C** (400 MHz, DMSO)


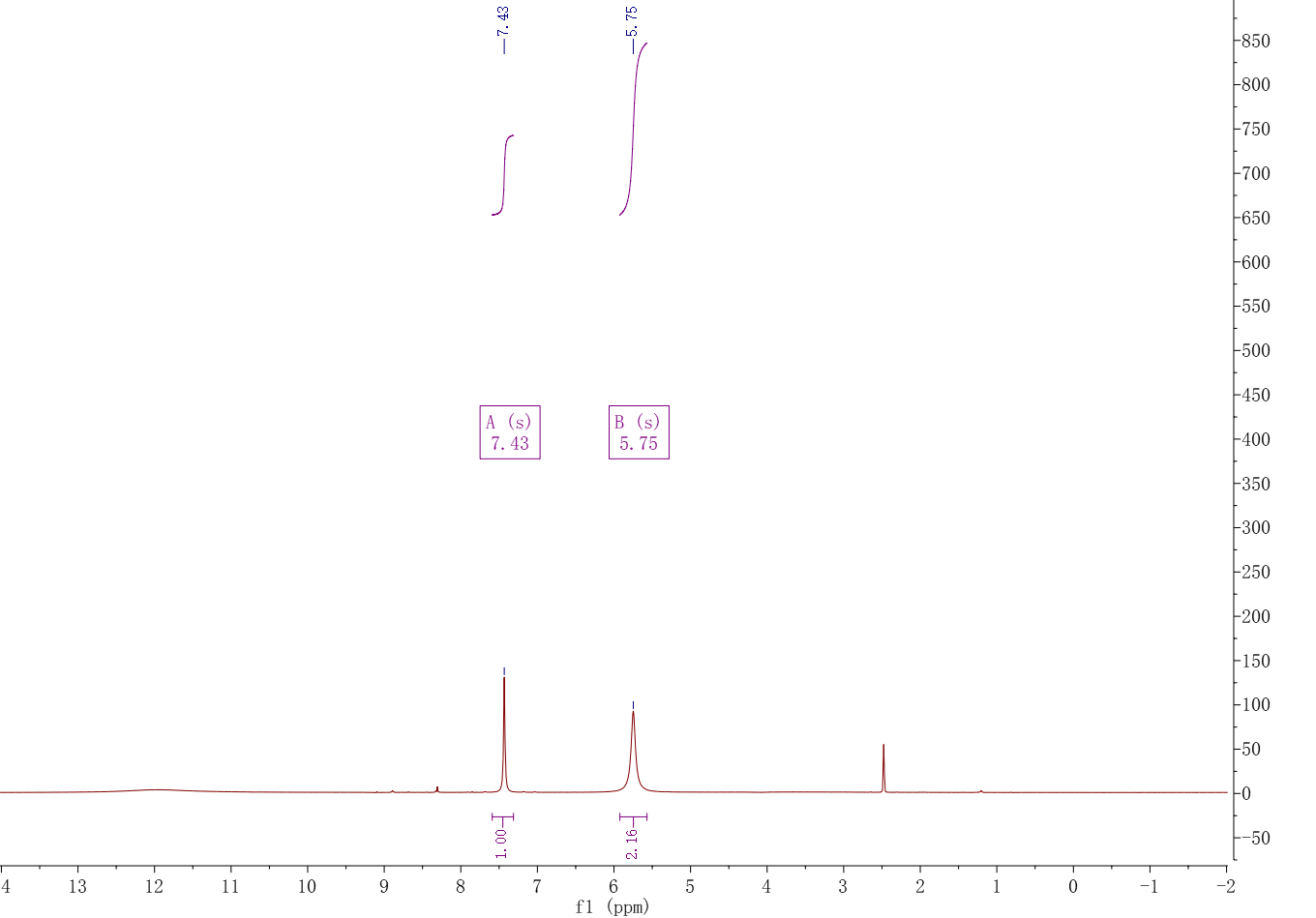


Fig. *^1^H NMR of* **D** (400 MHz, DMSO)


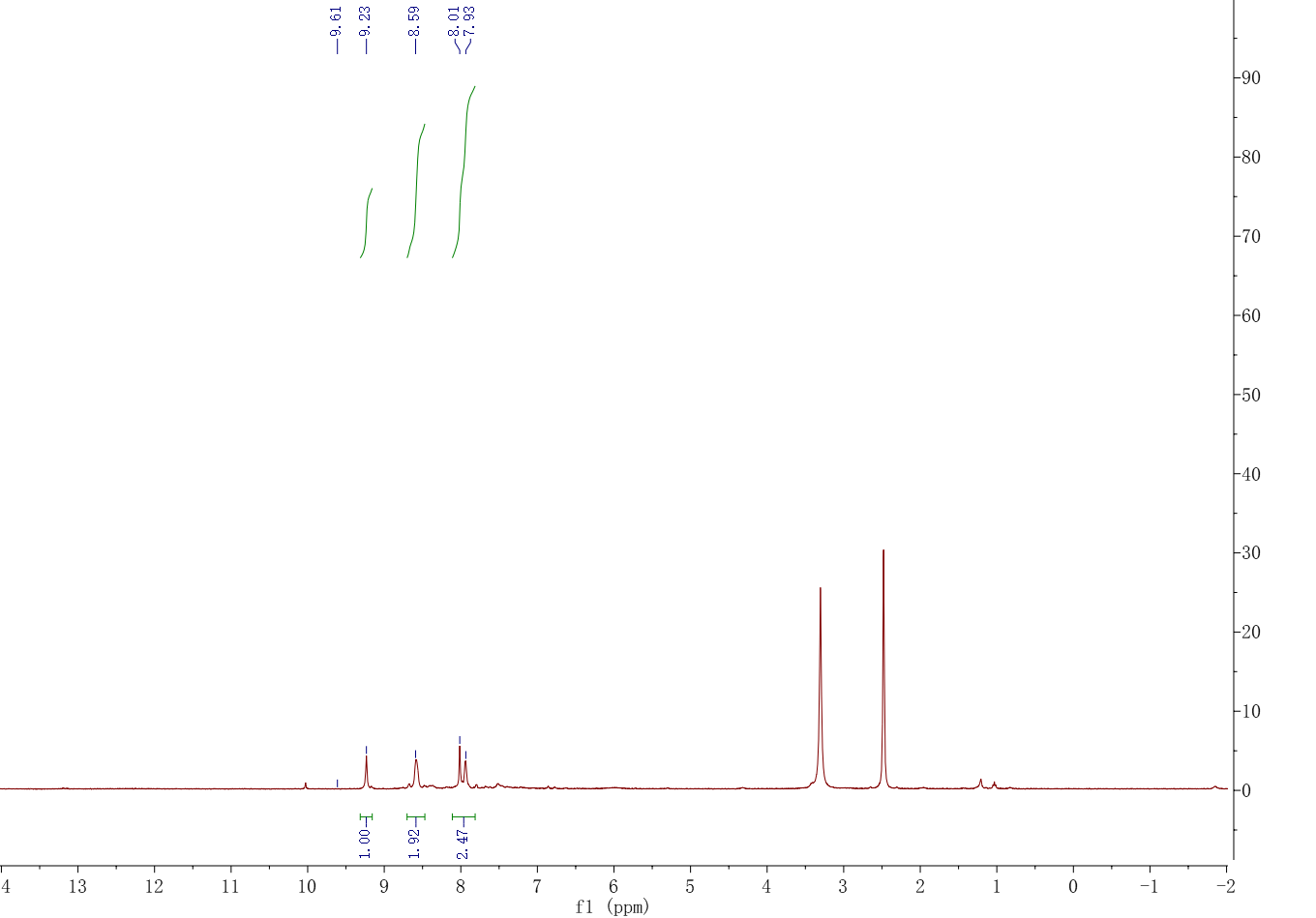


Fig. *^1^H NMR of* **E1** (400 MHz, DMSO)


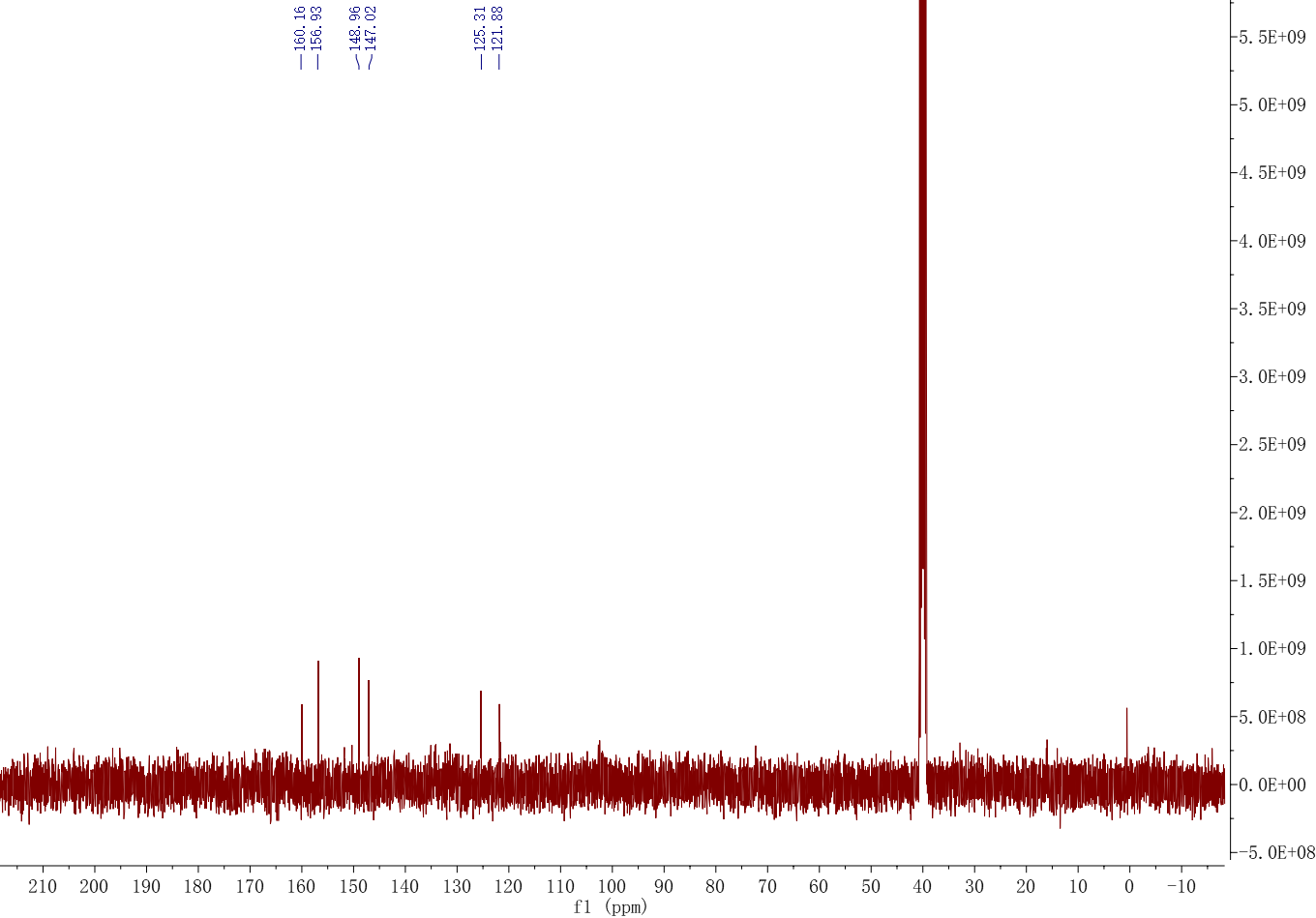


Fig. *^13^C NMR of* **E1** (100 MHz, DMSO)


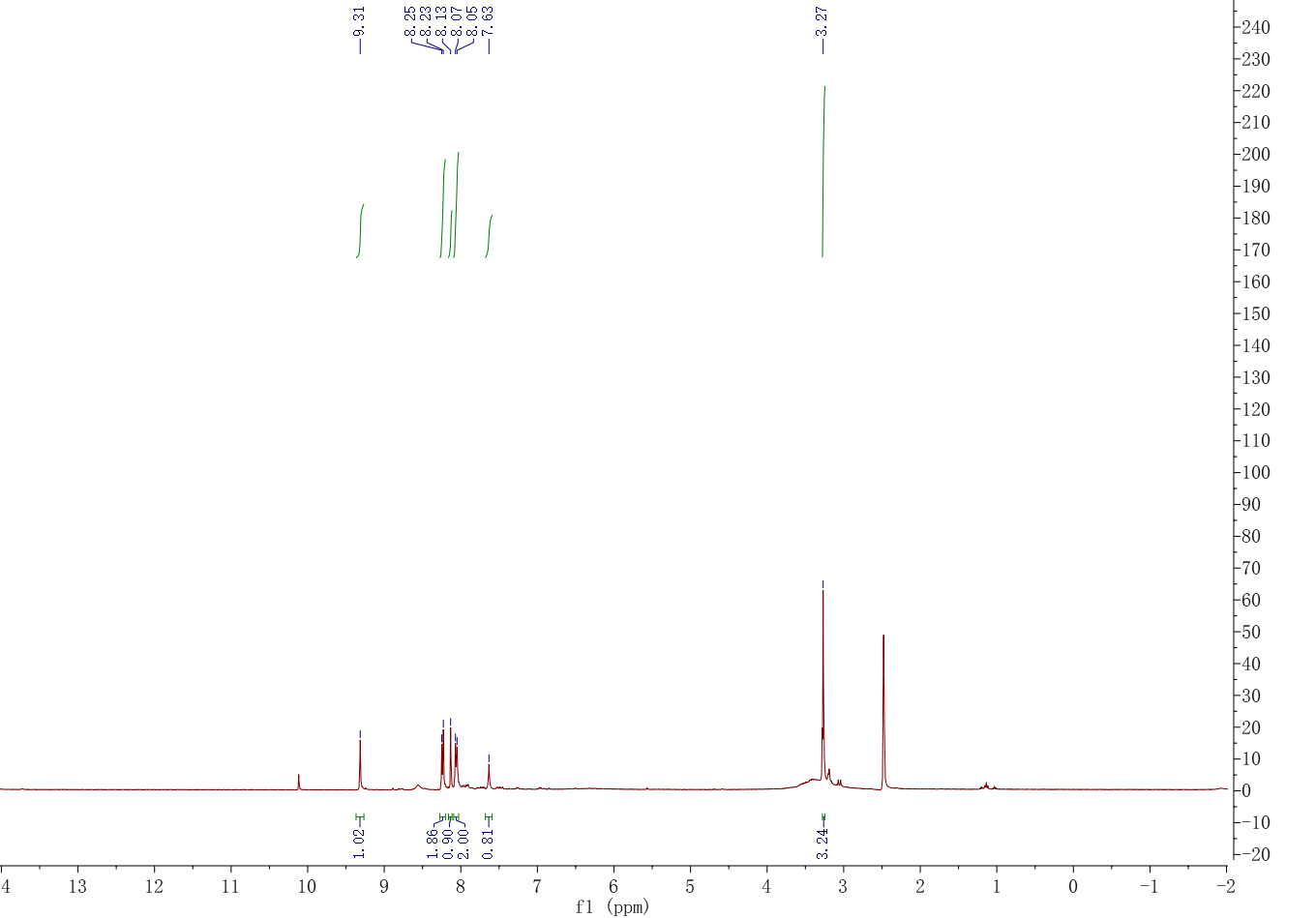


Fig. *^1^H NMR of* **E2** (400 MHz, DMSO)


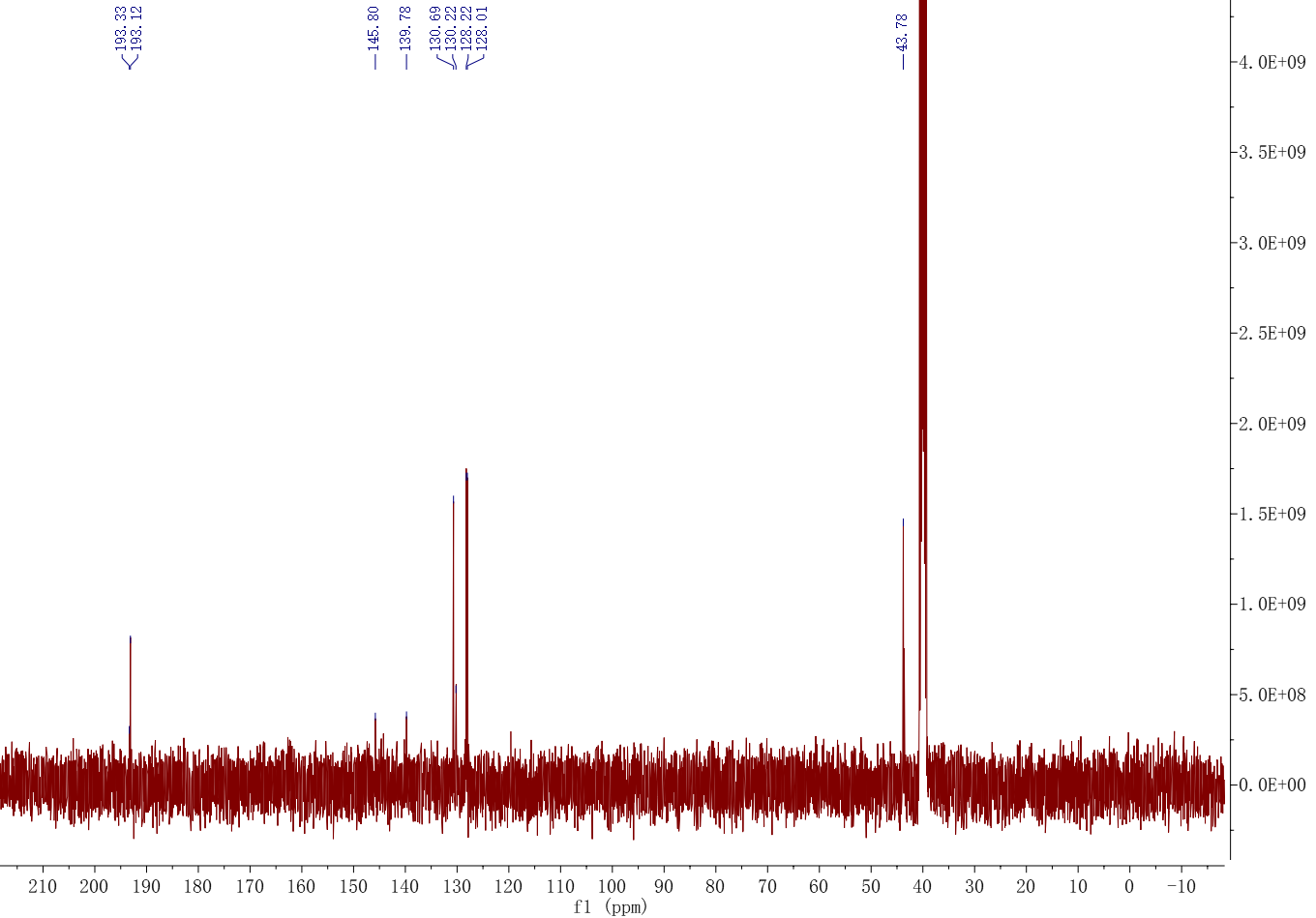


Fig. *^13^C NMR of* **E2** (100 MHz, DMSO)


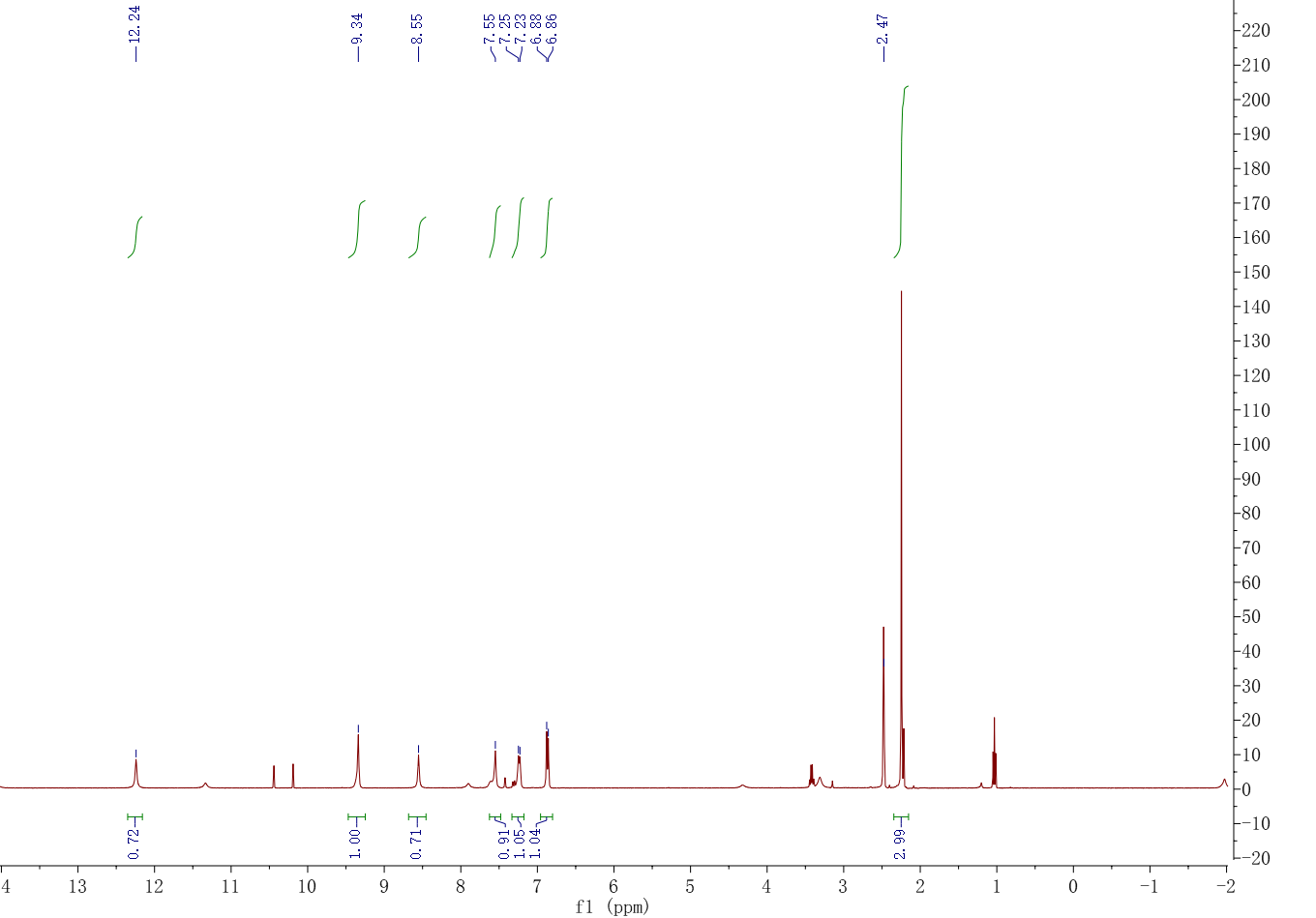


Fig. *^1^H NMR of* **E3** (400 MHz, DMSO)


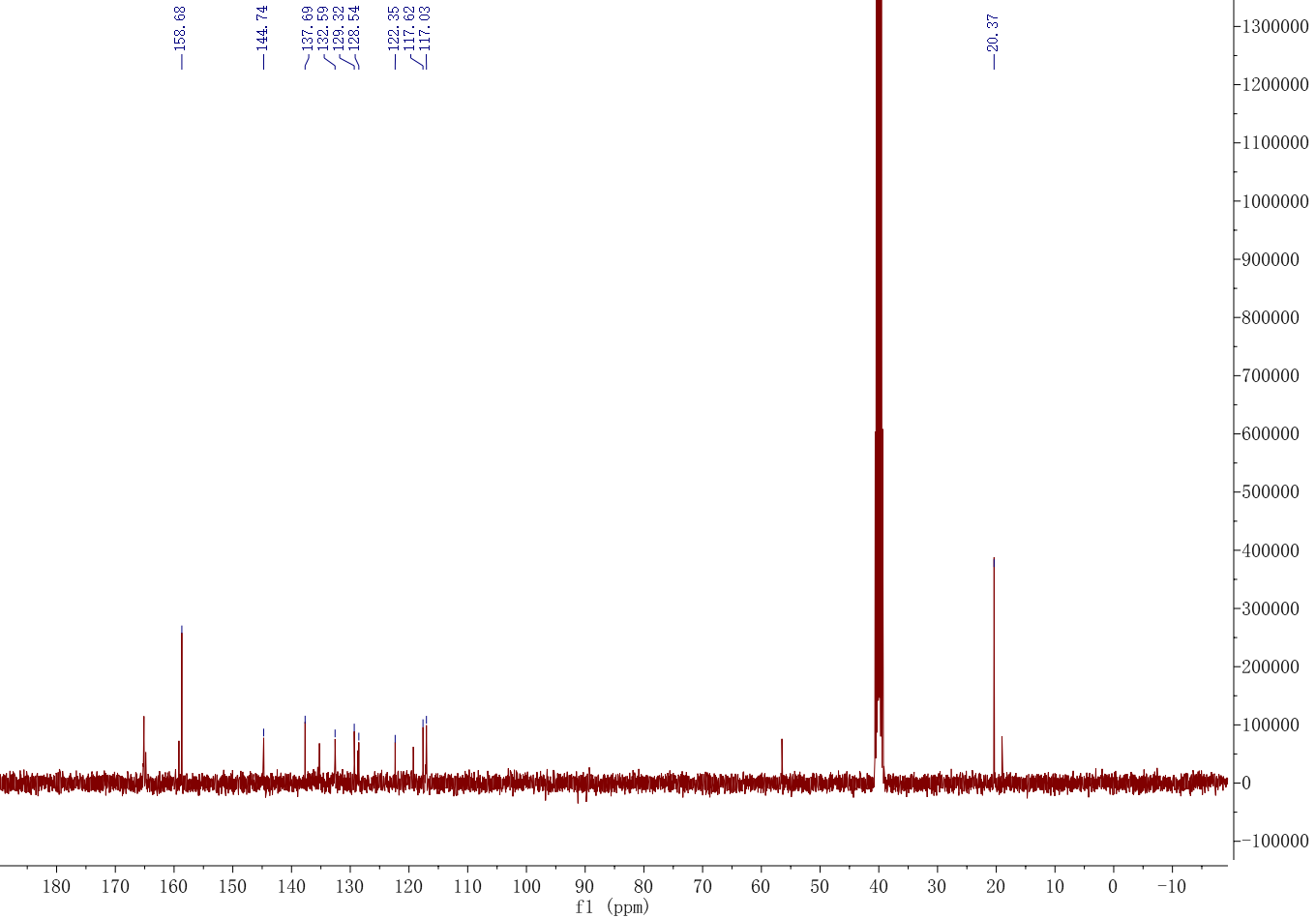


Fig. *^13^C NMR of* **E3** (100 MHz, DMSO)


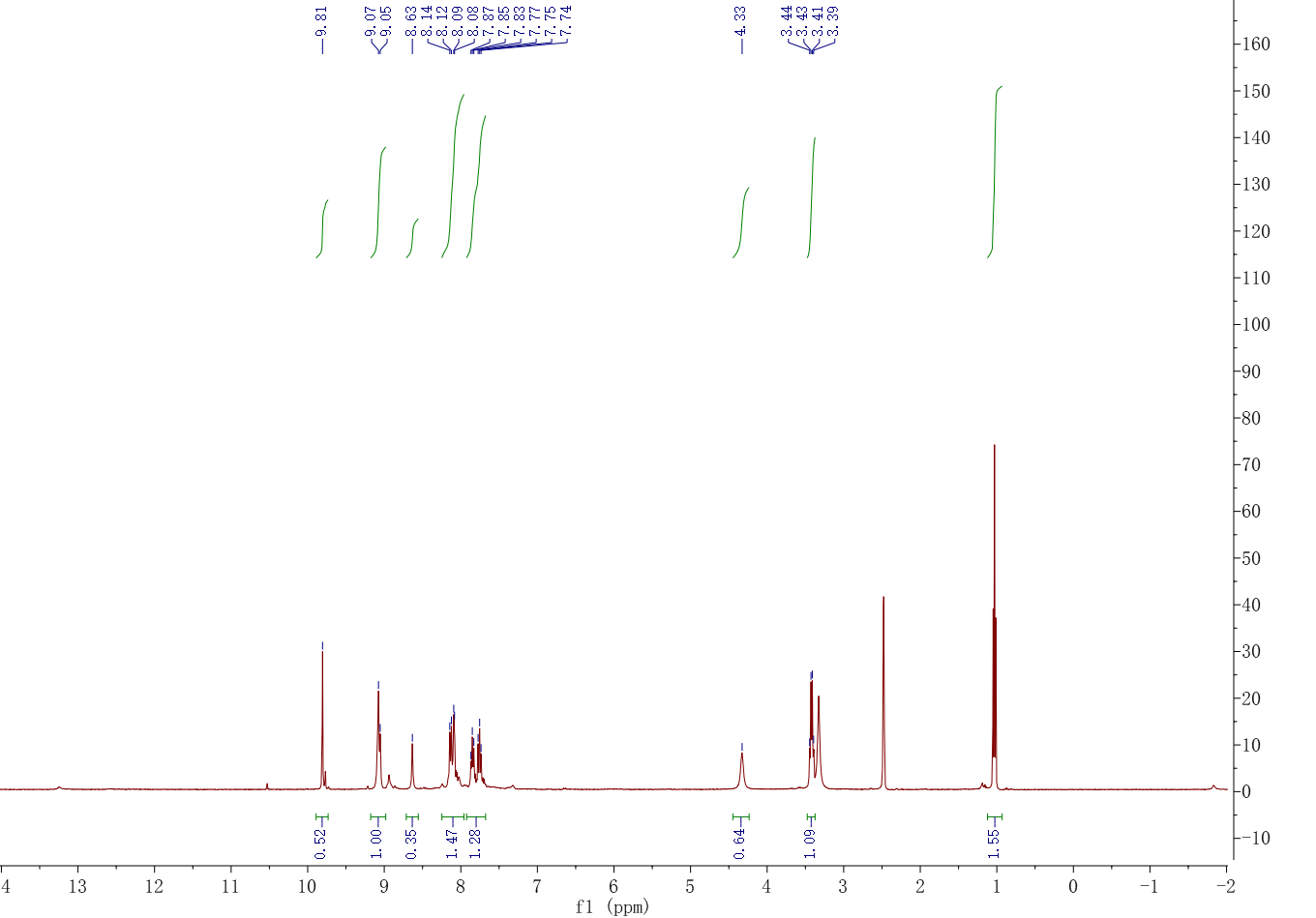


Fig. *^1^H NMR of* **E4** (400 MHz, DMSO)


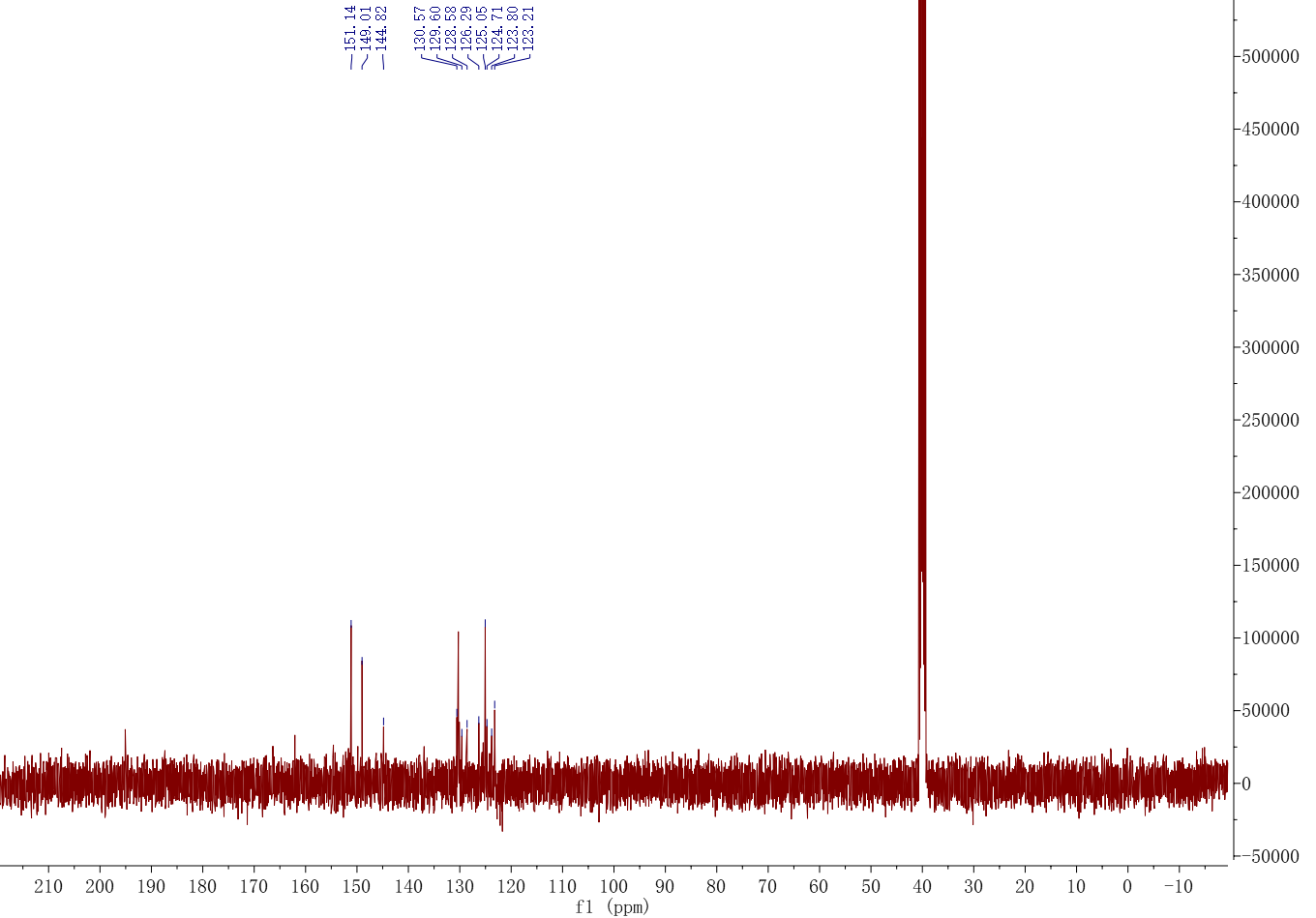


Fig. *^13^C NMR of* **E4** (100 MHz, DMSO)


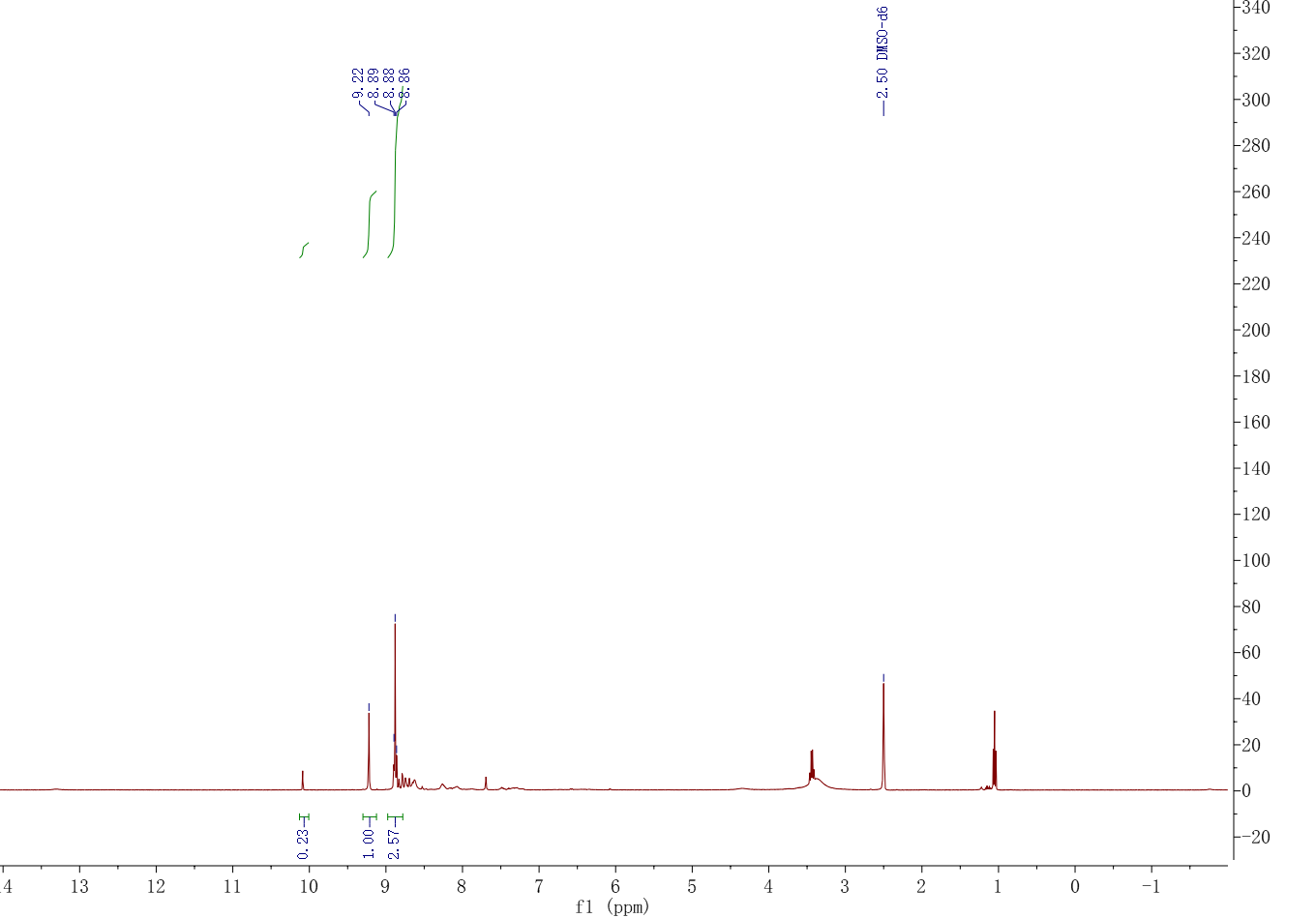


Fig. *^1^H NMR of* **E5** (400 MHz, DMSO)


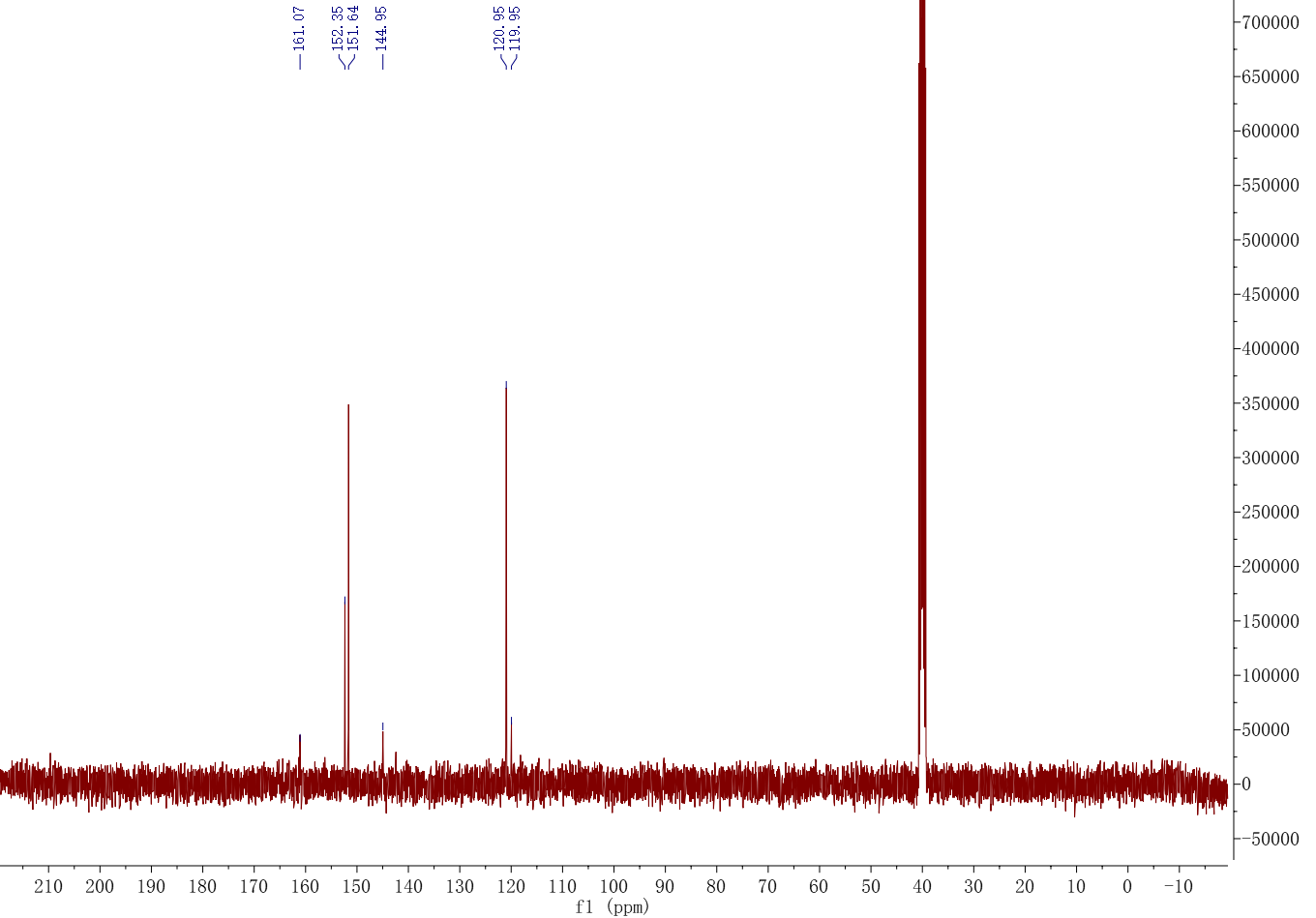


Fig. *^13^C NMR of* **E5** (100 MHz, DMSO)


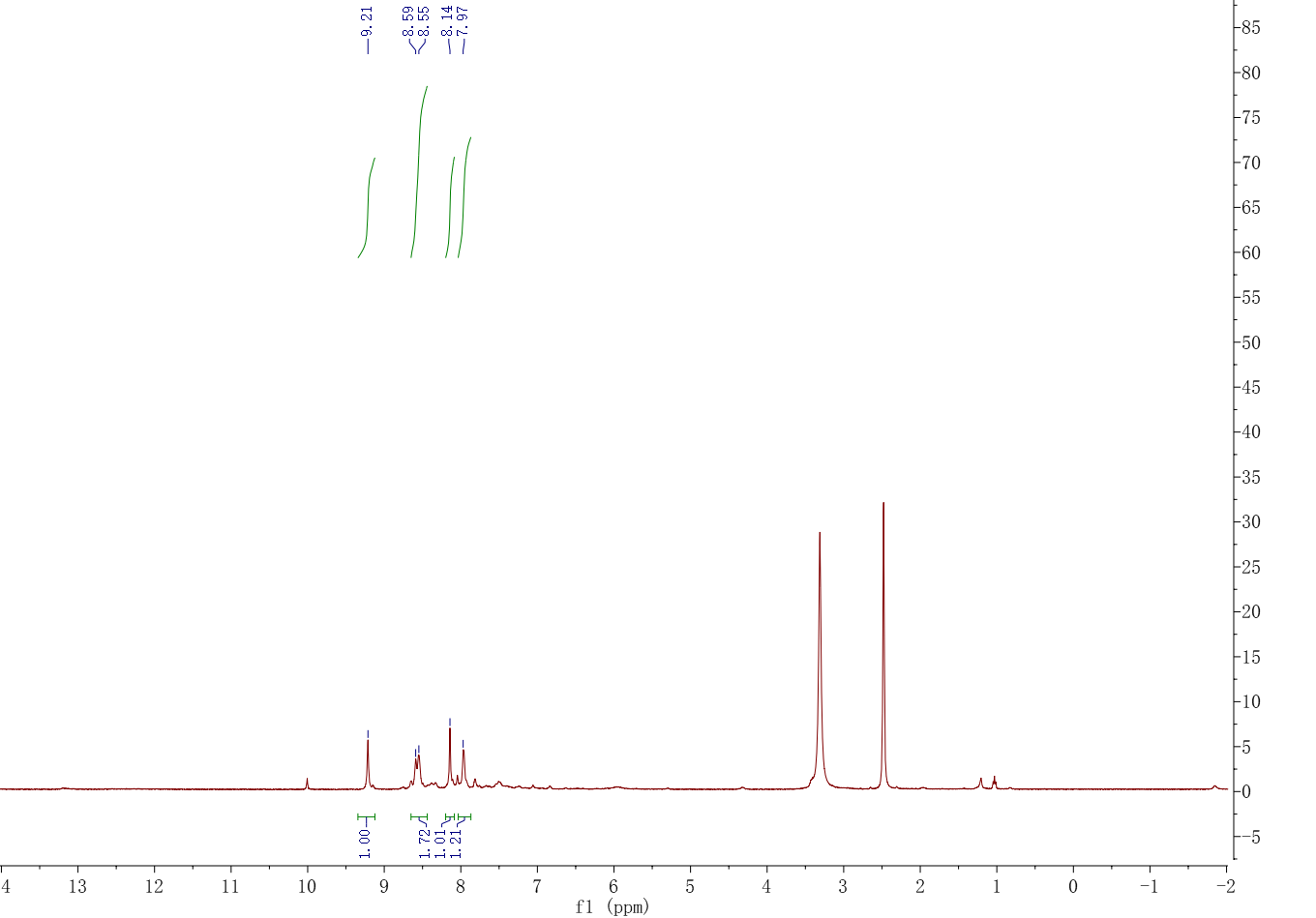


Fig. *^1^H NMR of* **E6** (400 MHz, DMSO)


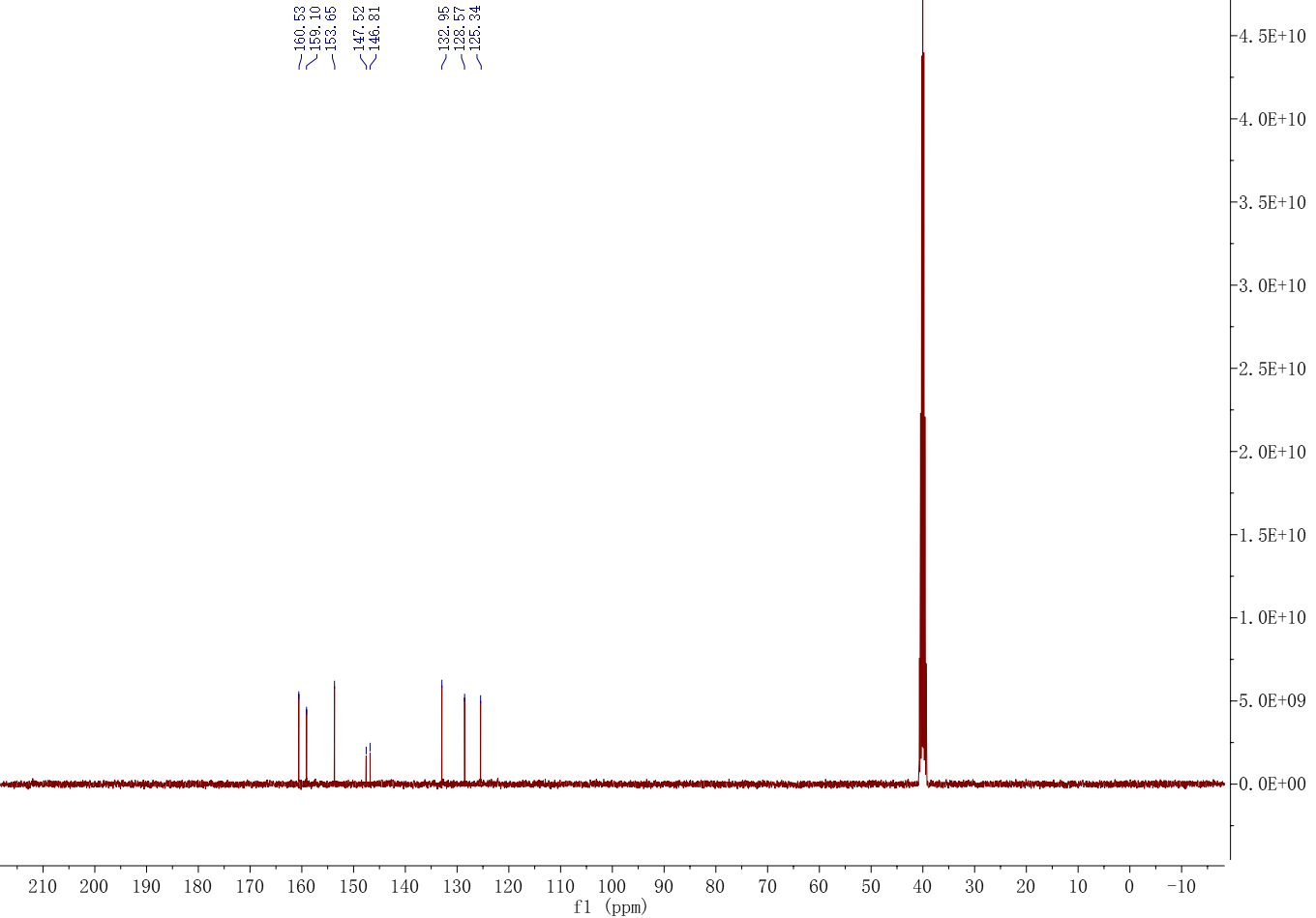


Fig. *^13^C NMR of* **E6** (100 MHz, DMSO)


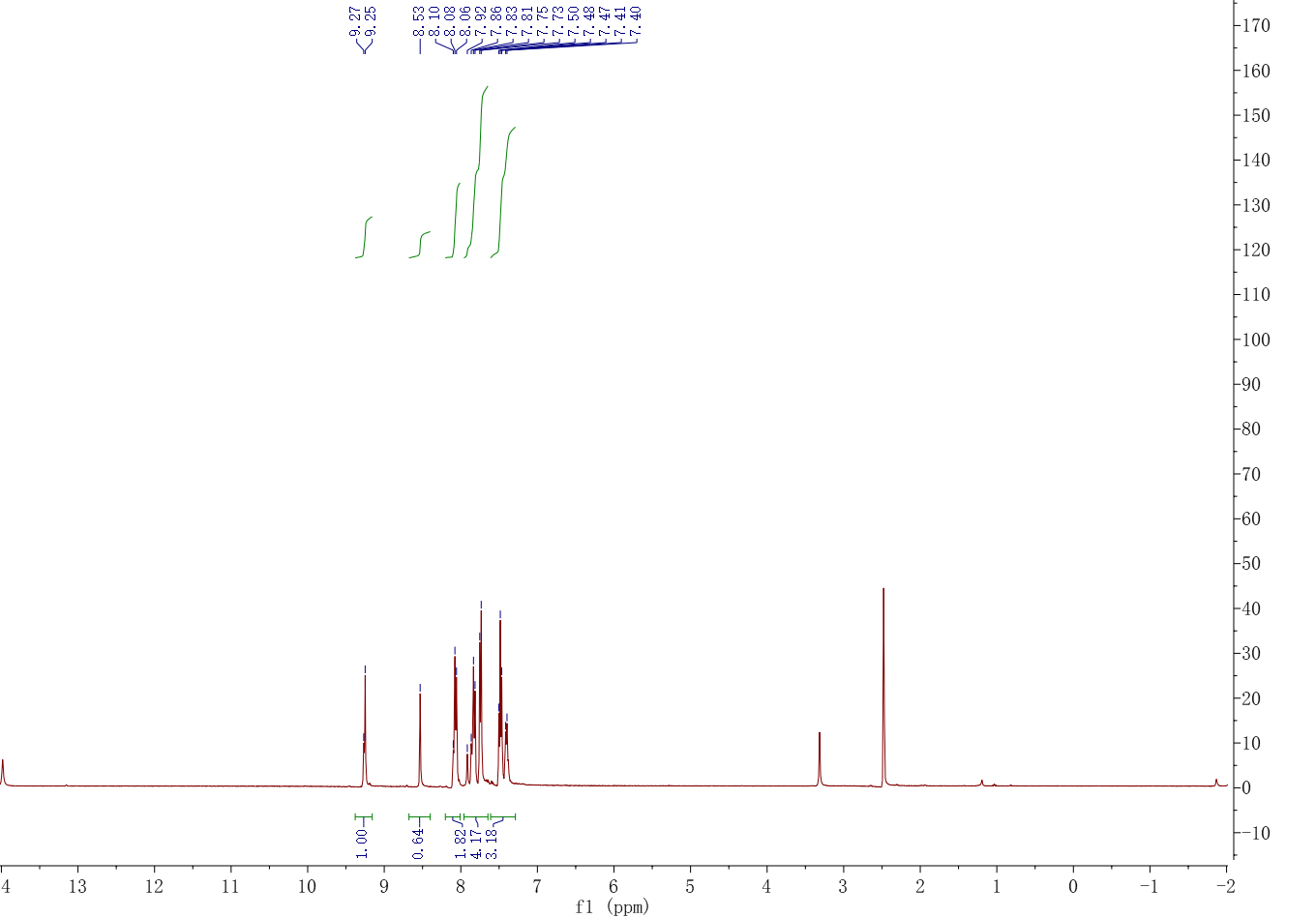


Fig. *^1^H NMR of* **E7** (400 MHz, DMSO)


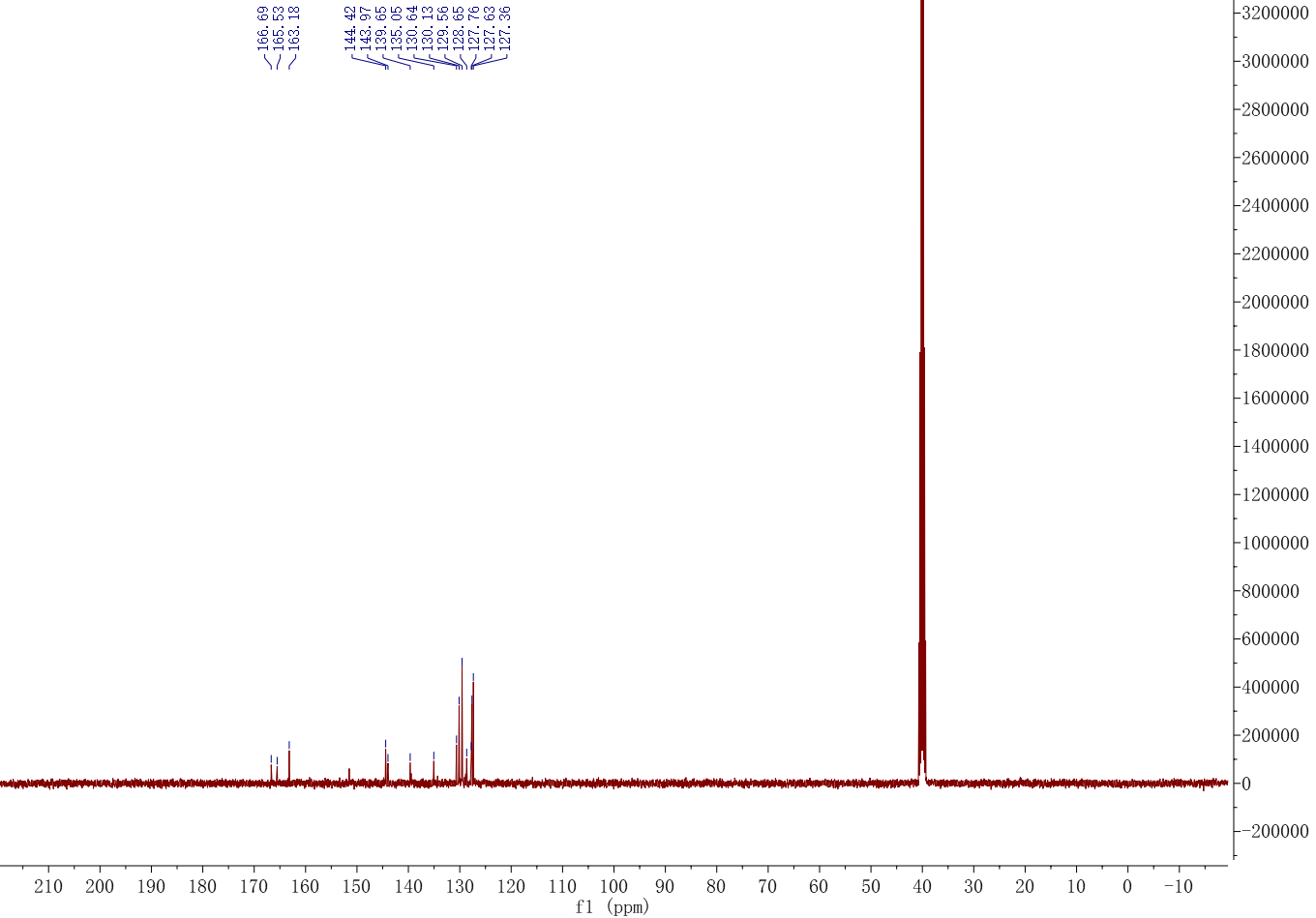


Fig. *^13^C NMR of* **E7** (100 MHz, DMSO)


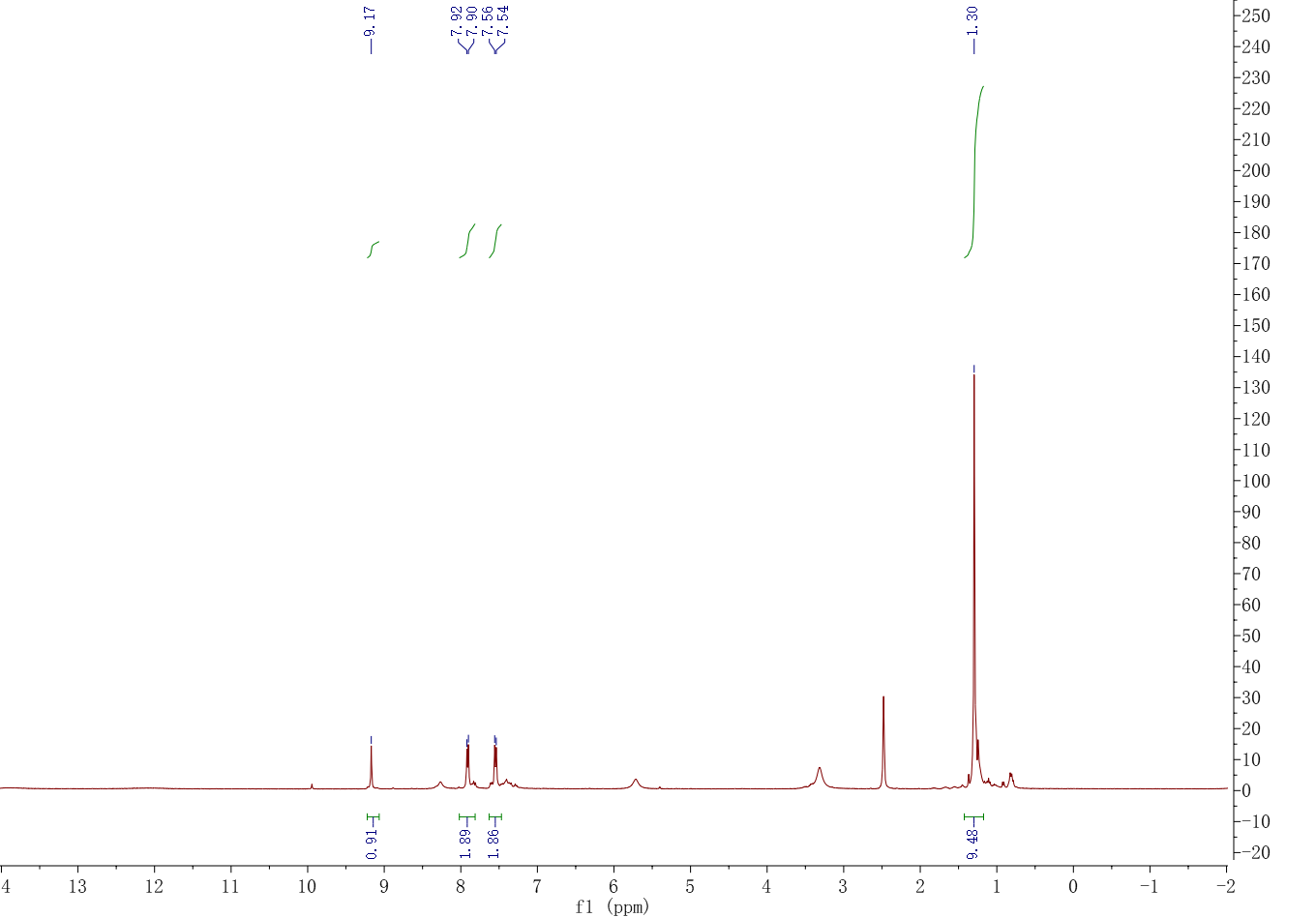


Fig. *^1^H NMR of* **E8** (400 MHz, DMSO)


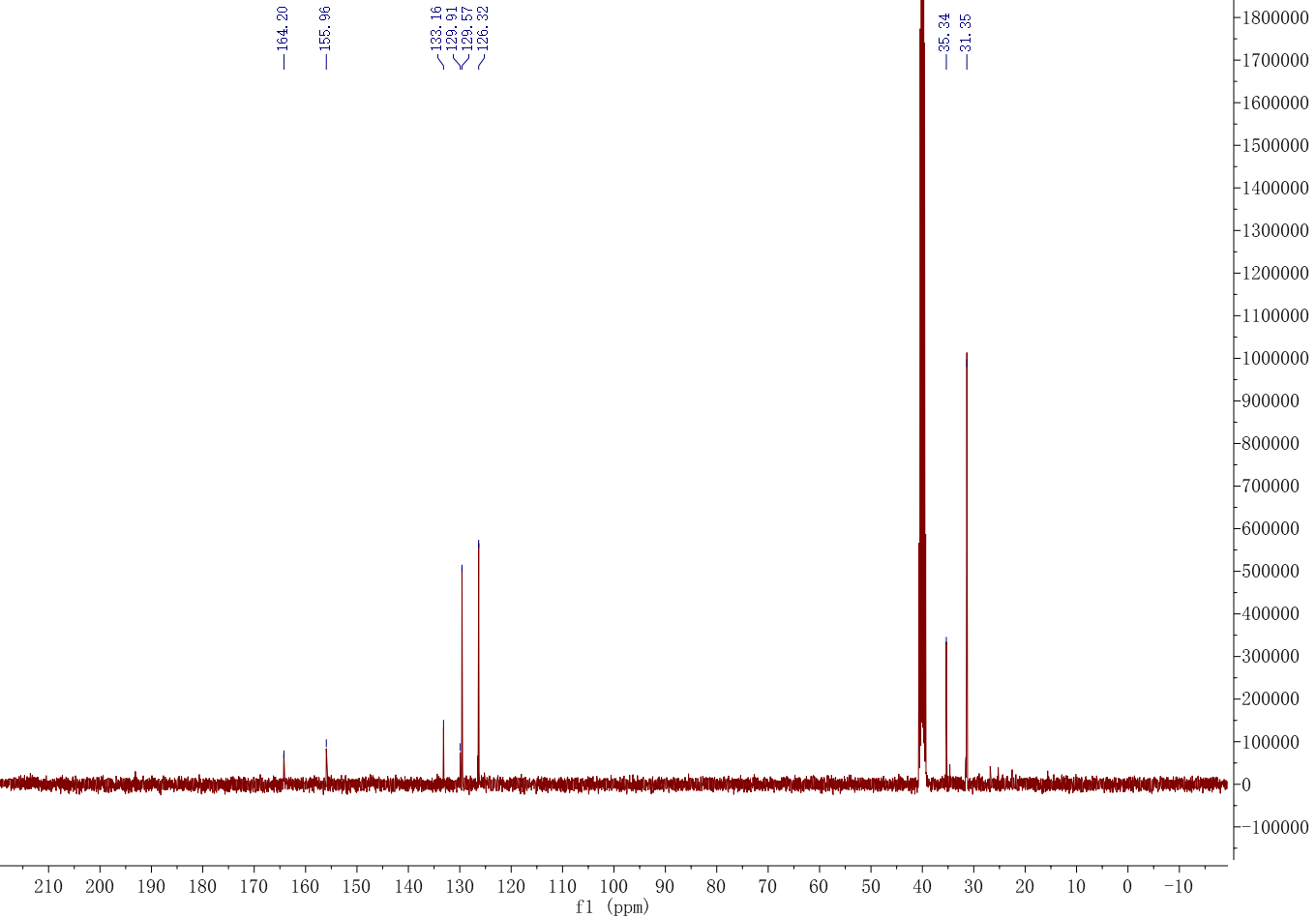


Fig. *^13^C NMR of* **E8** (100 MHz, DMSO)


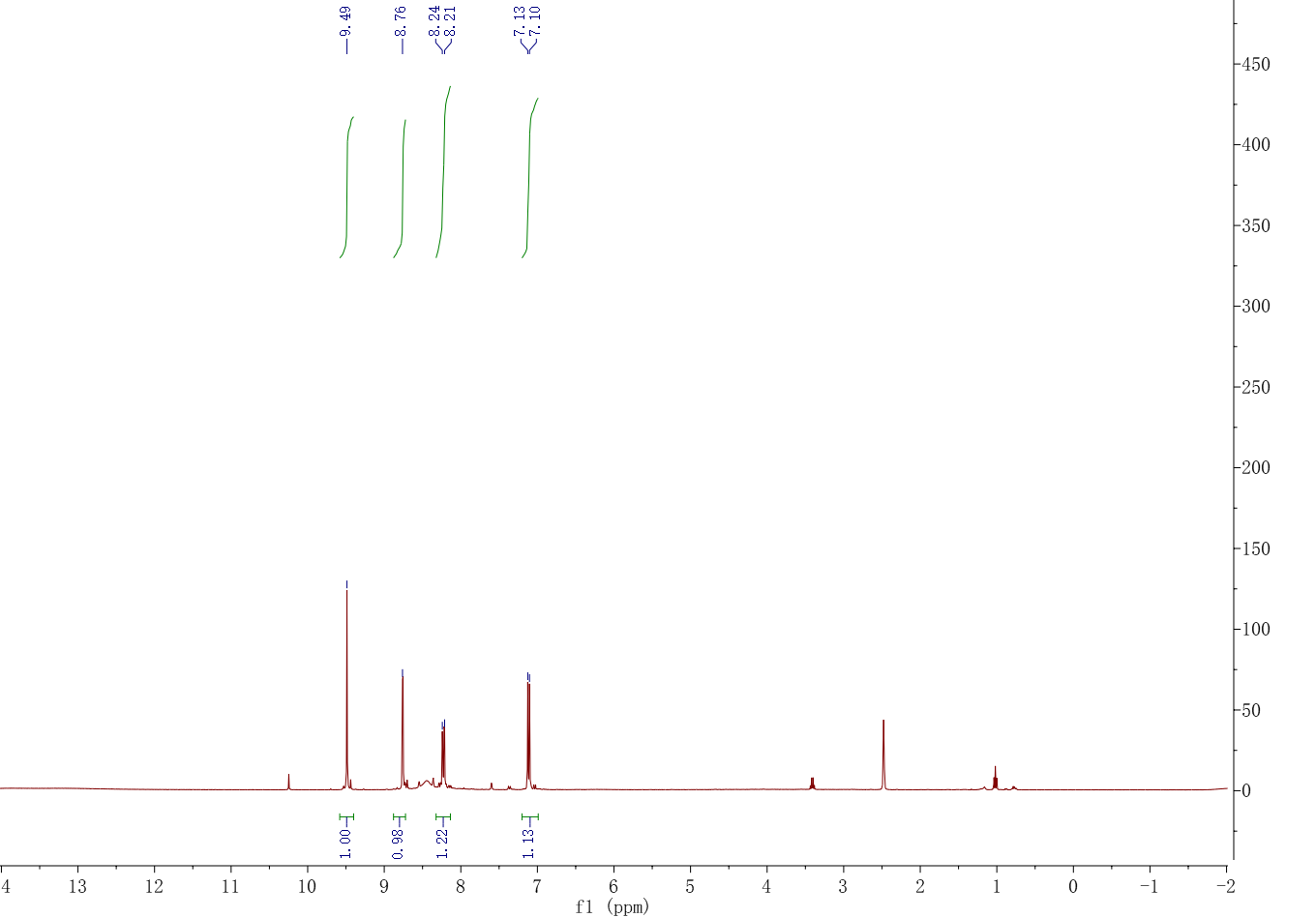


Fig. *^1^H NMR of* **E9** (400 MHz, DMSO)


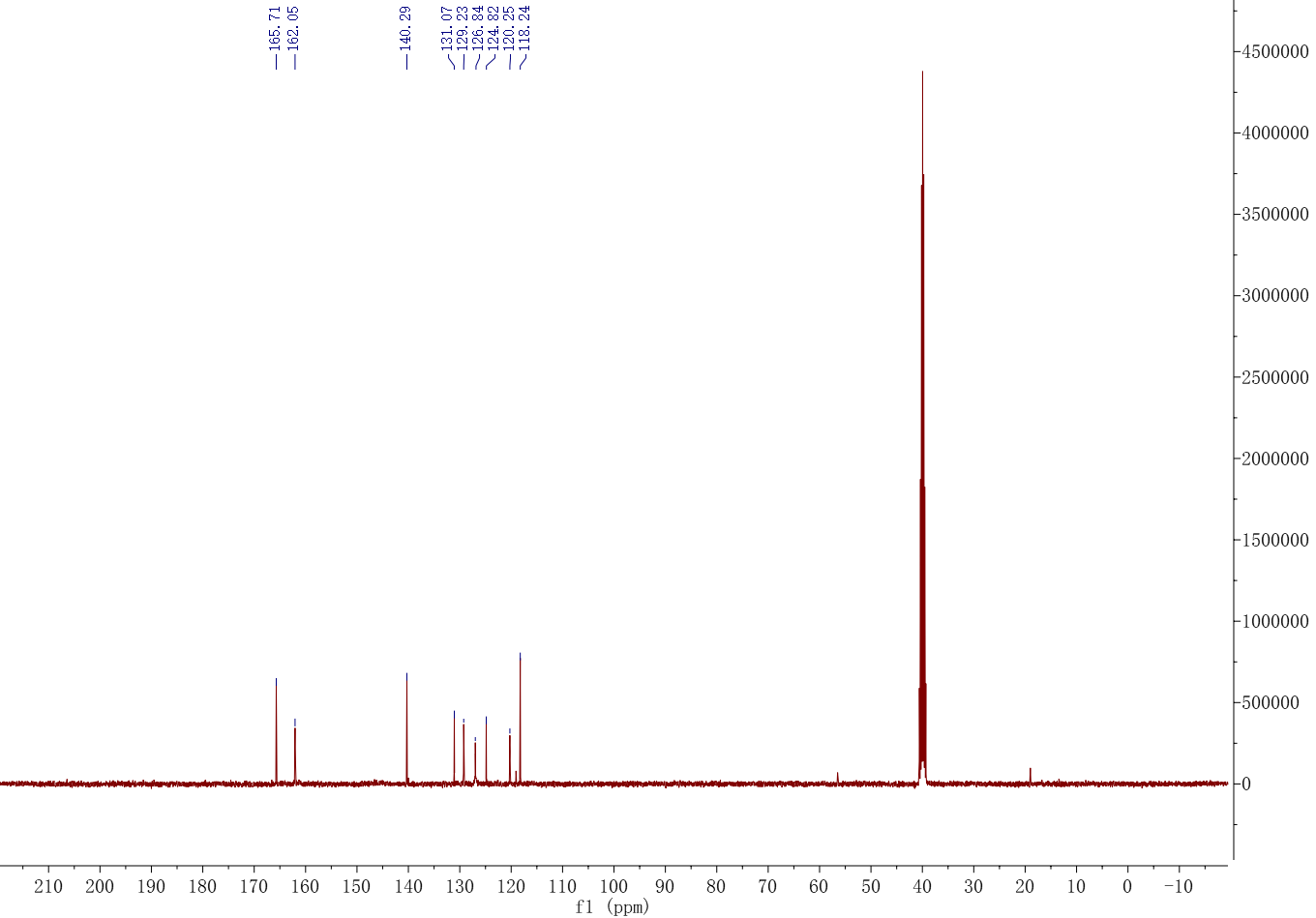


Fig. *^13^C NMR of* **E9** (100 MHz, DMSO)


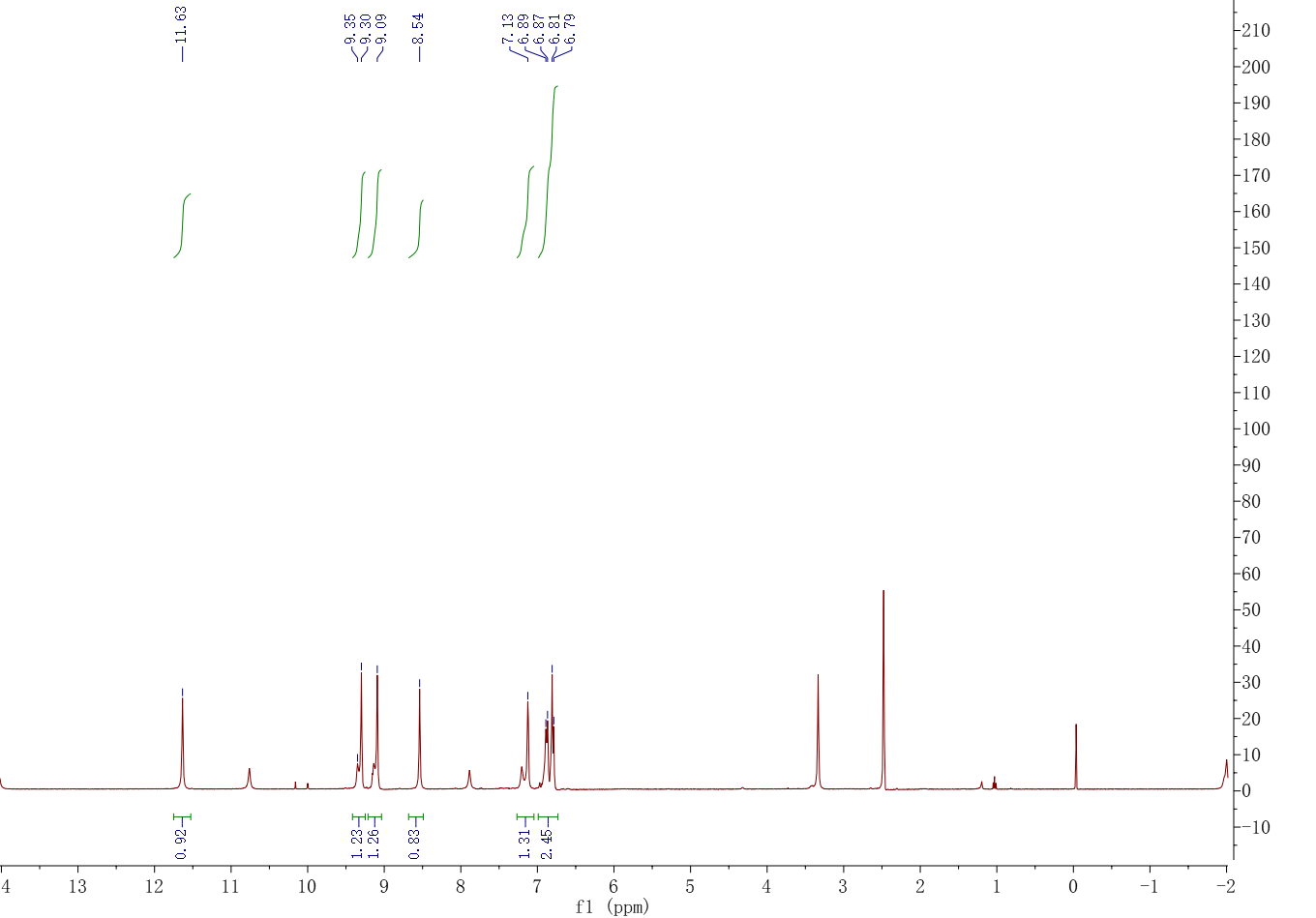


Fig. *^1^H NMR of* **E10** (400 MHz, DMSO)


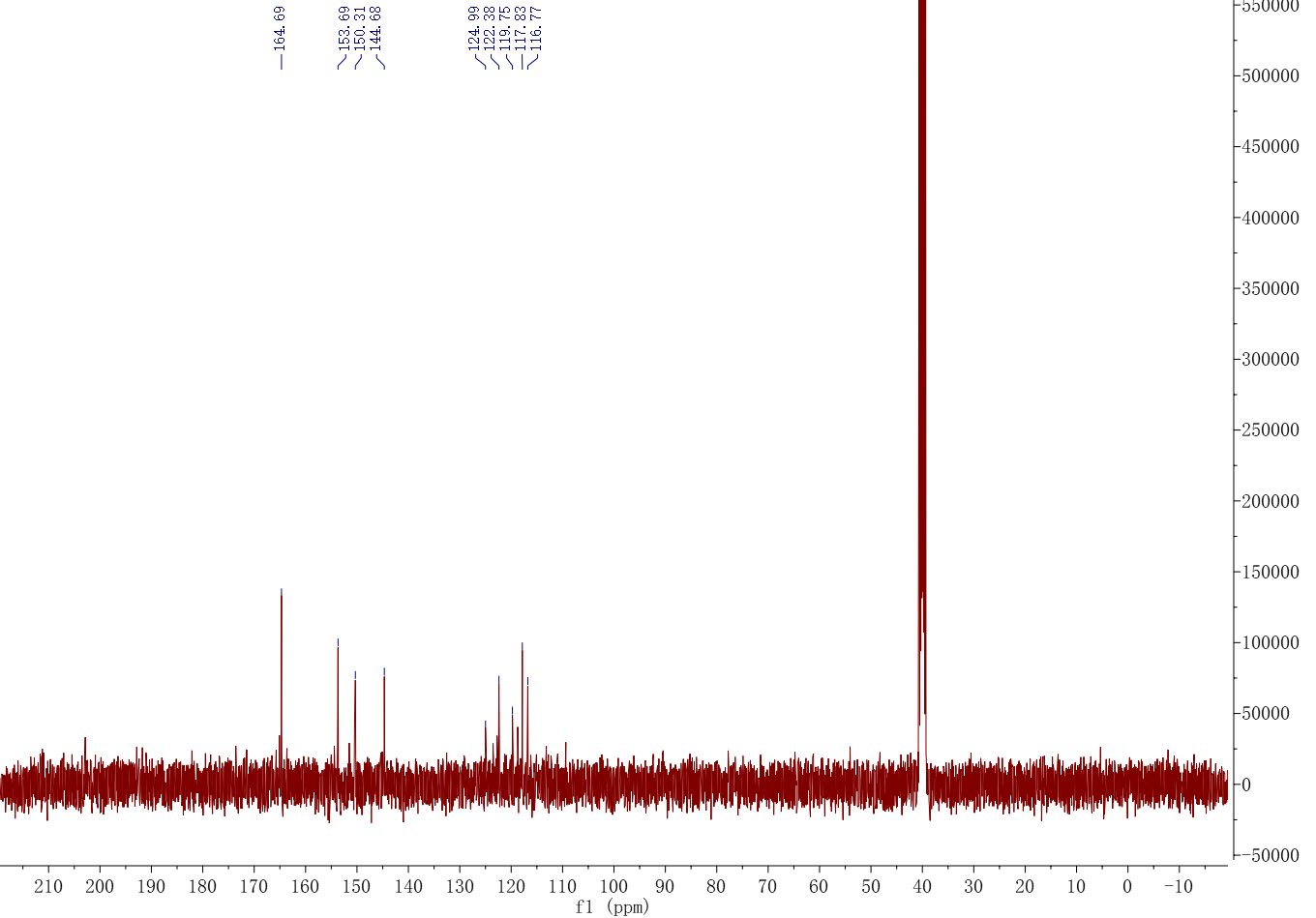


Fig. *^13^C NMR of* **E10** (100 MHz, DMSO)


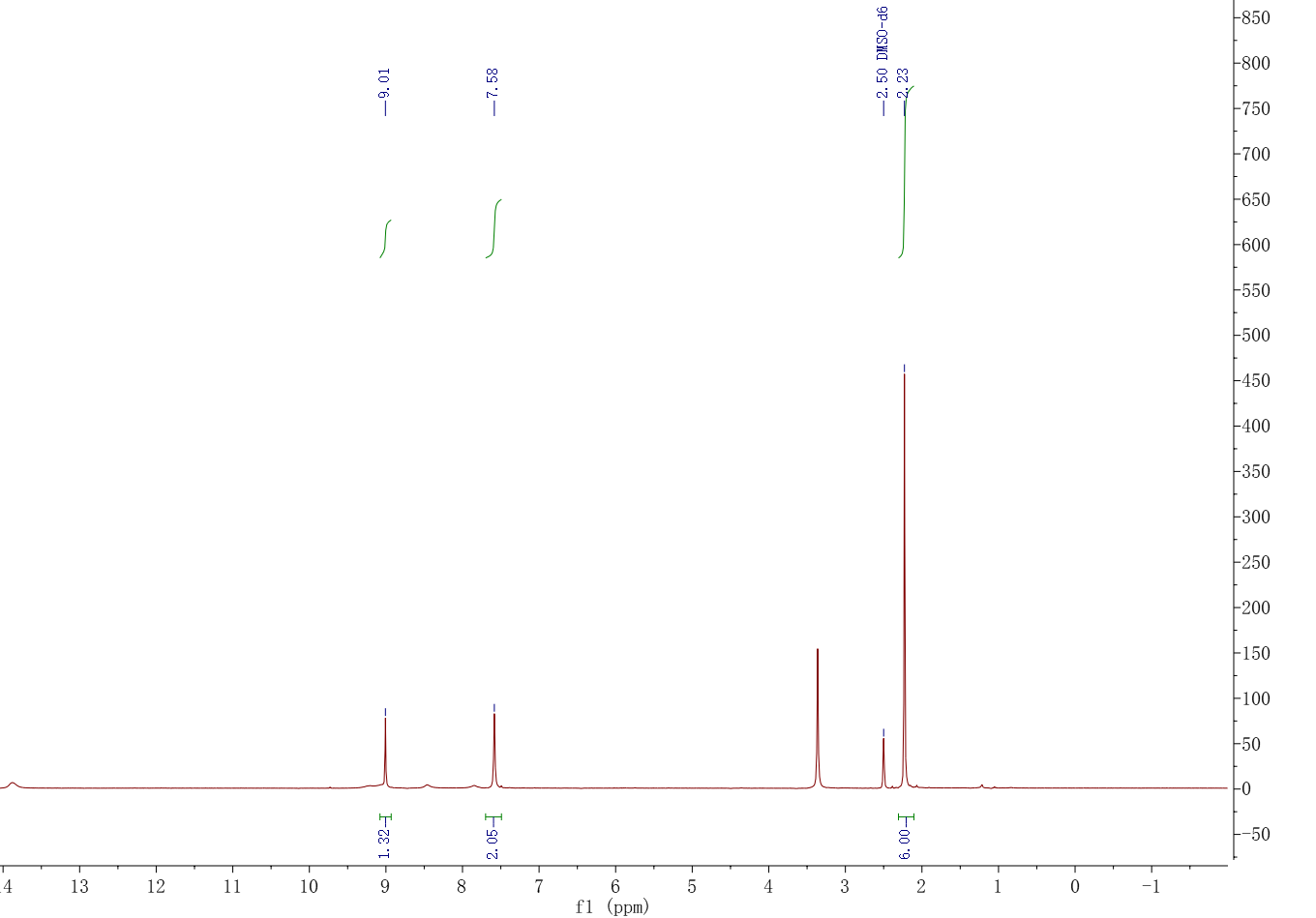


Fig. *^1^H NMR of* **E11** (400 MHz, DMSO)


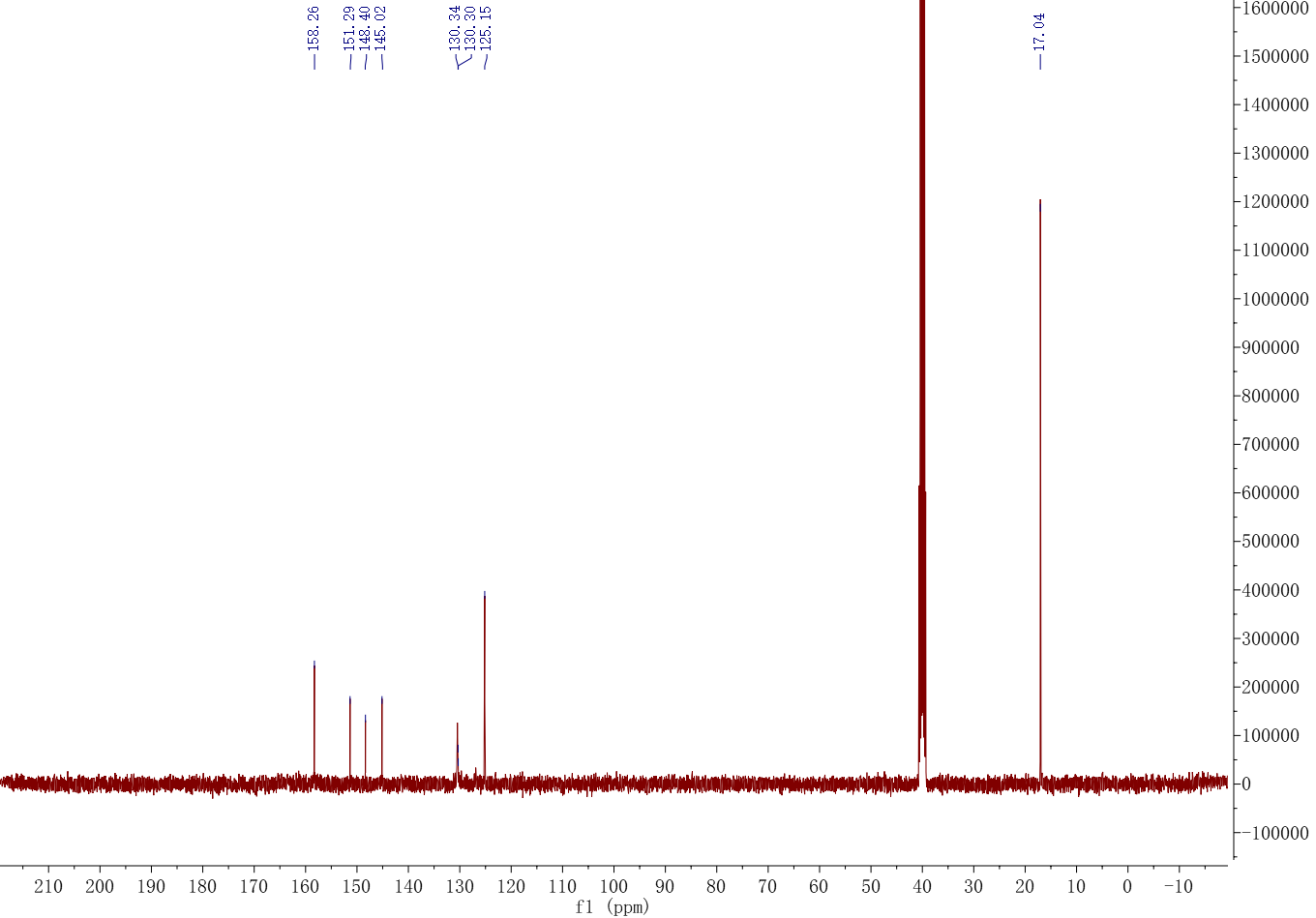


Fig. *^13^C NMR of* **E11** (100 MHz, DMSO)


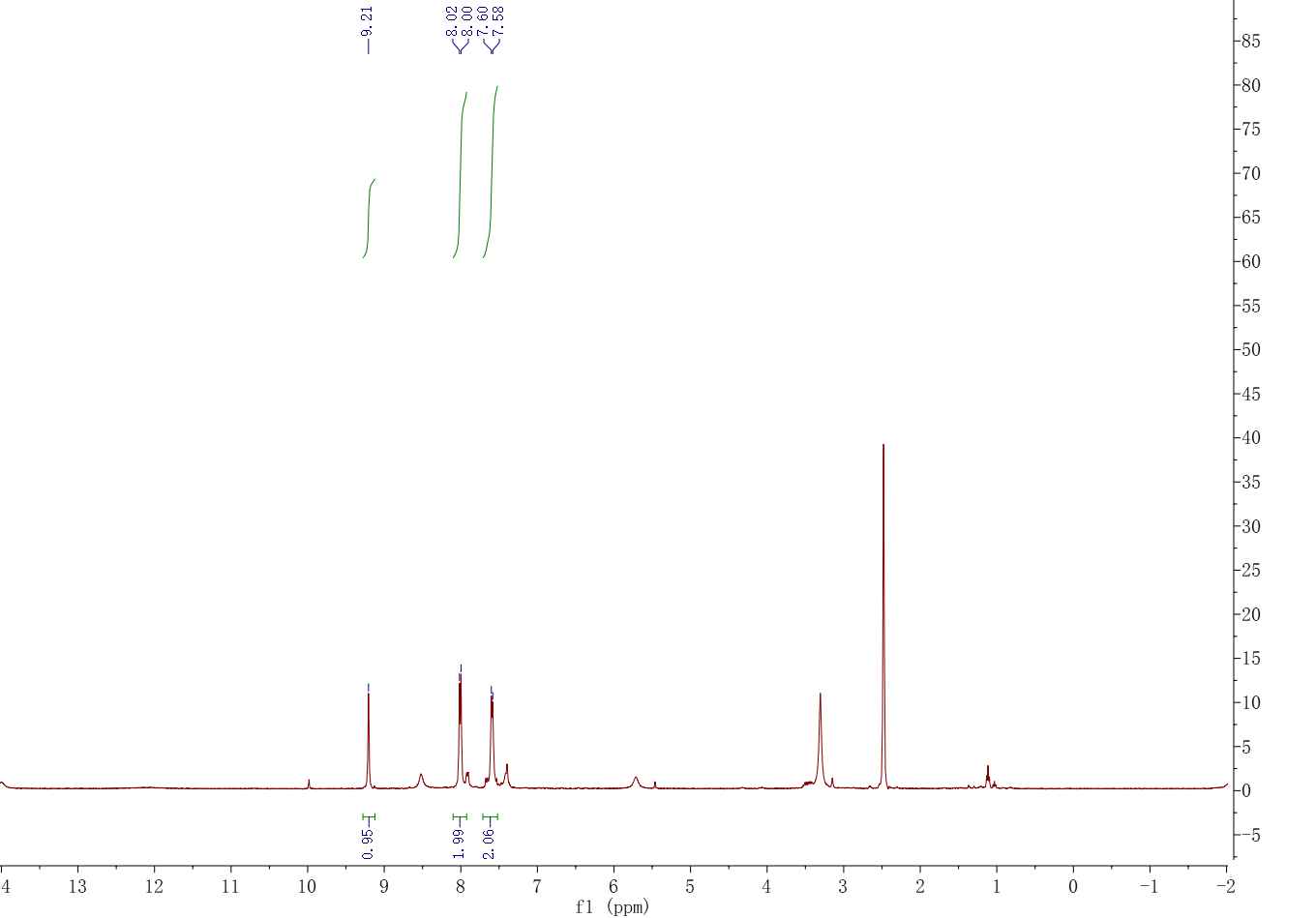


Fig. *^1^H NMR of* **E12** (400 MHz, DMSO)


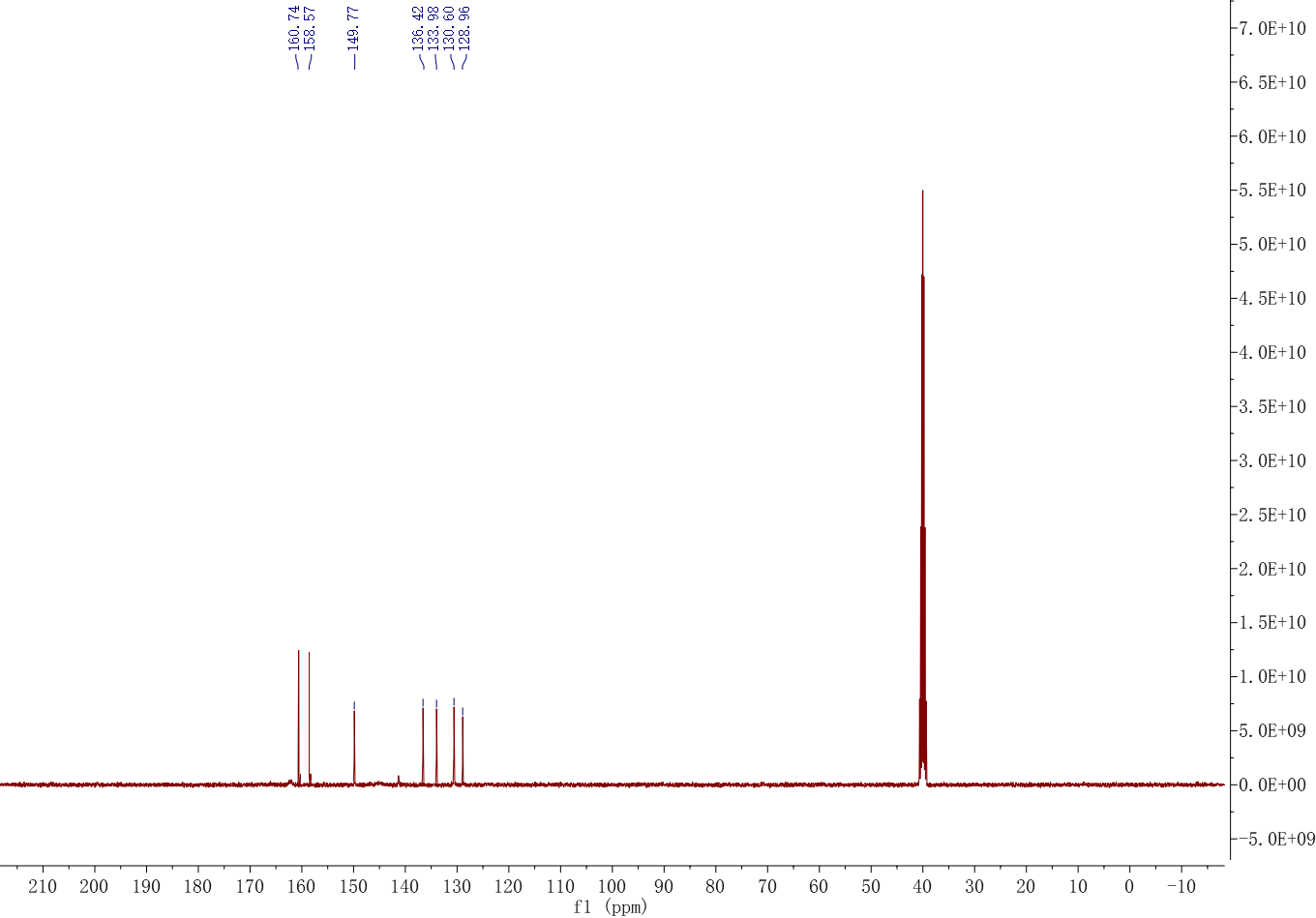


Fig. *^13^C NMR of* **E12** (100 MHz, DMSO)


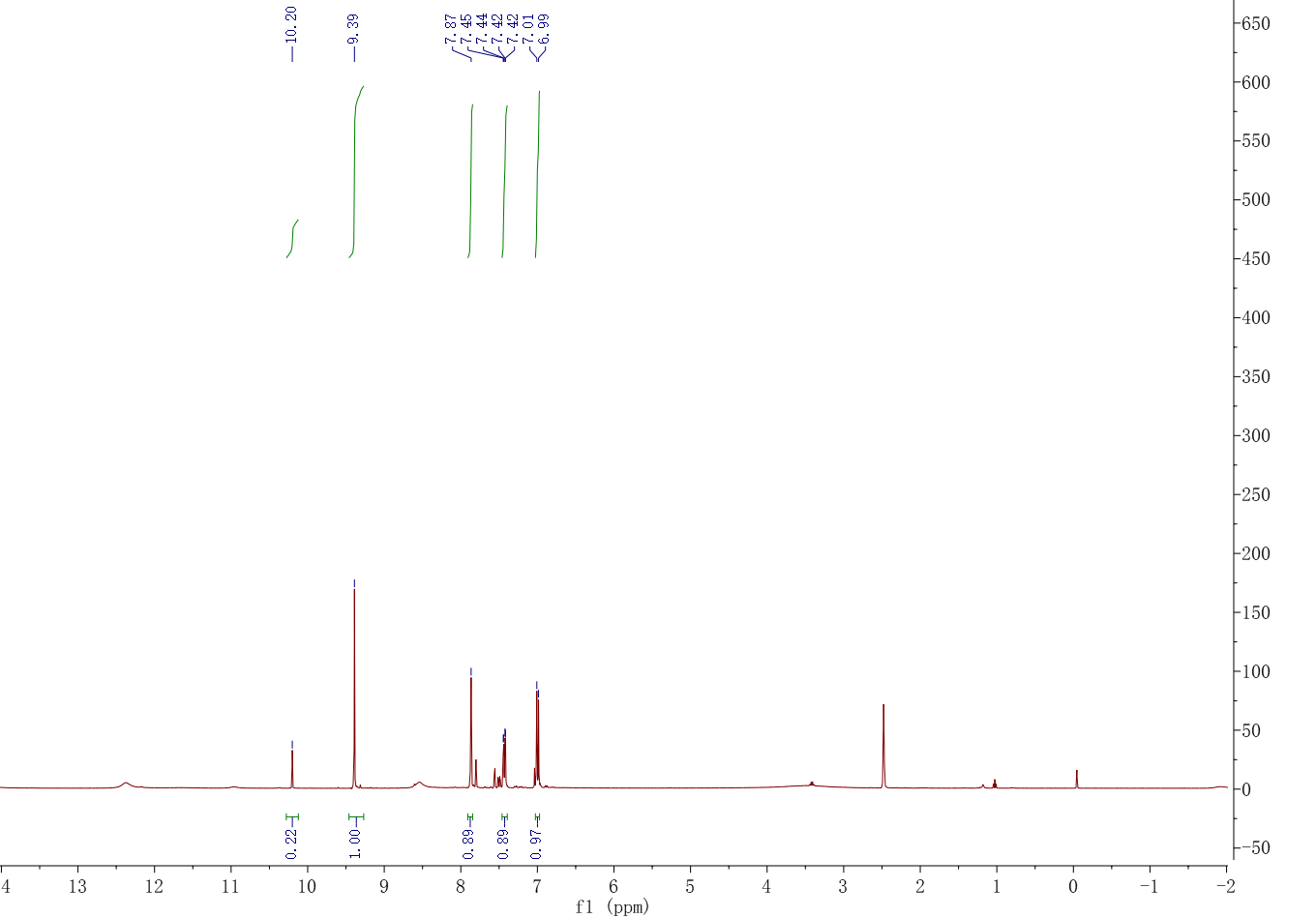


Fig. *^1^H NMR of* **E13** (400 MHz, DMSO)


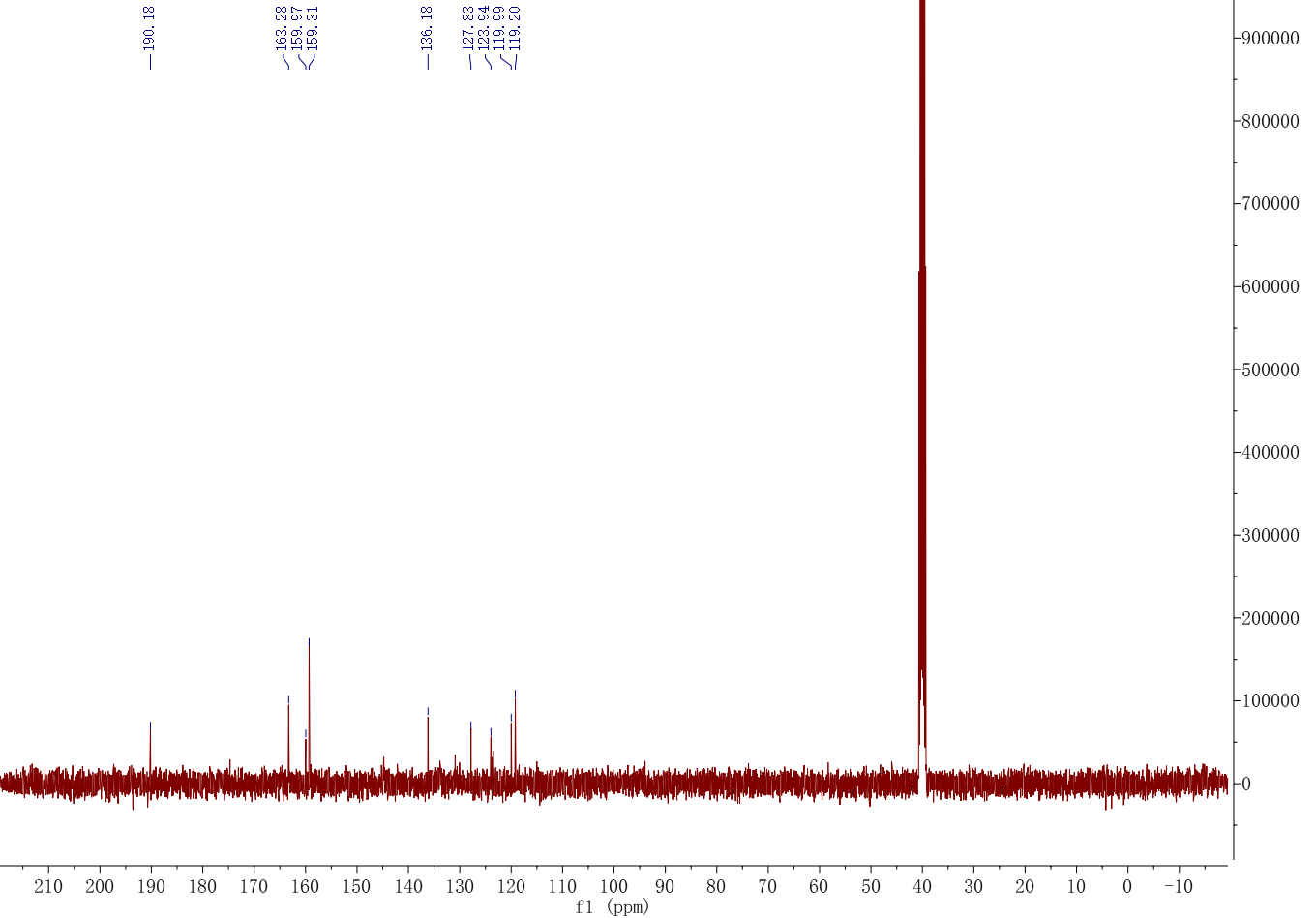


Fig. *^13^C NMR of* **E13** (100 MHz, DMSO)


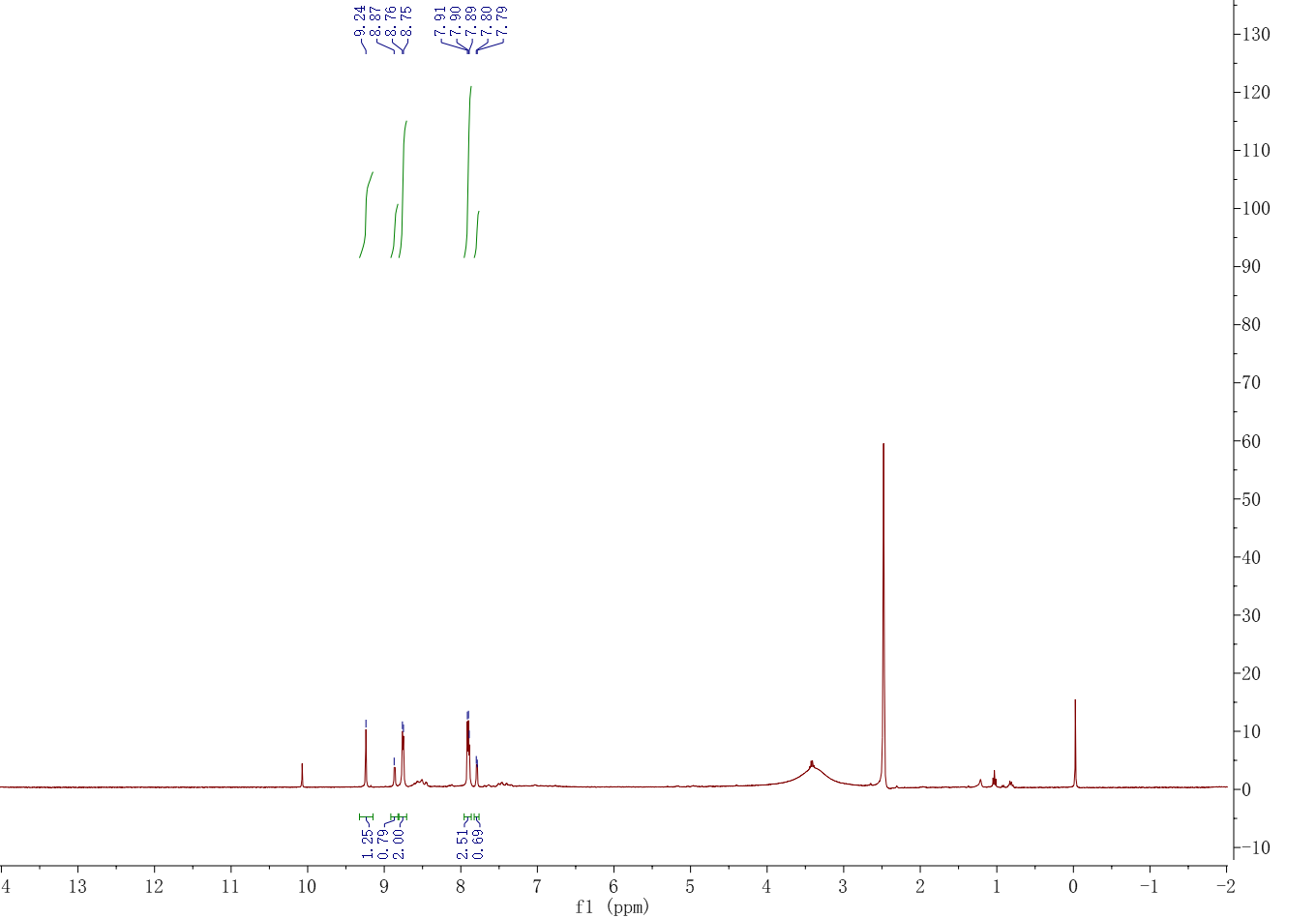


Fig. *^1^H NMR of* **E14** (400 MHz, DMSO)


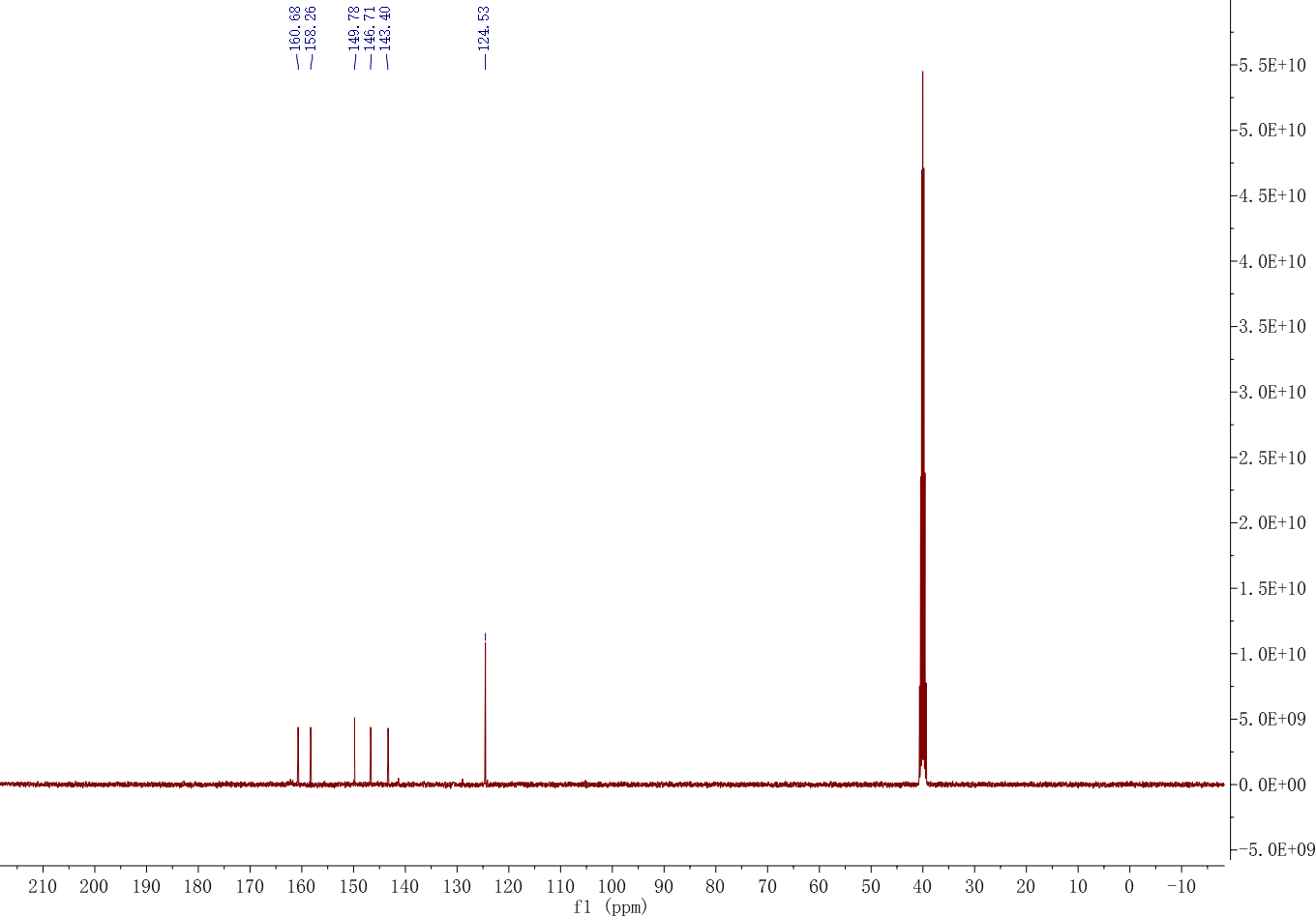


Fig. *^13^C NMR of* **E14** (100 MHz, DMSO)


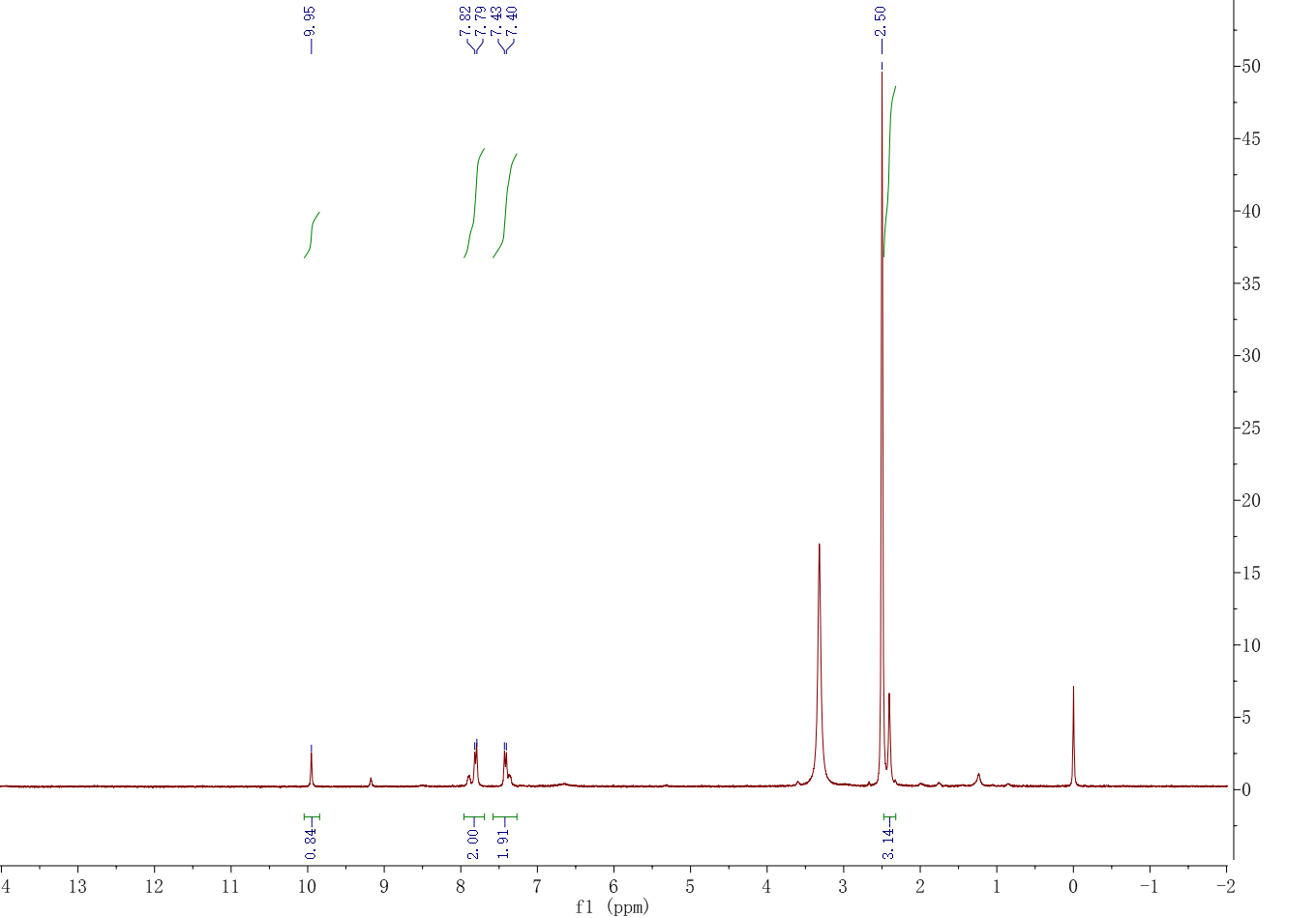


Fig. *^1^H NMR of* **E15** (400 MHz, DMSO)


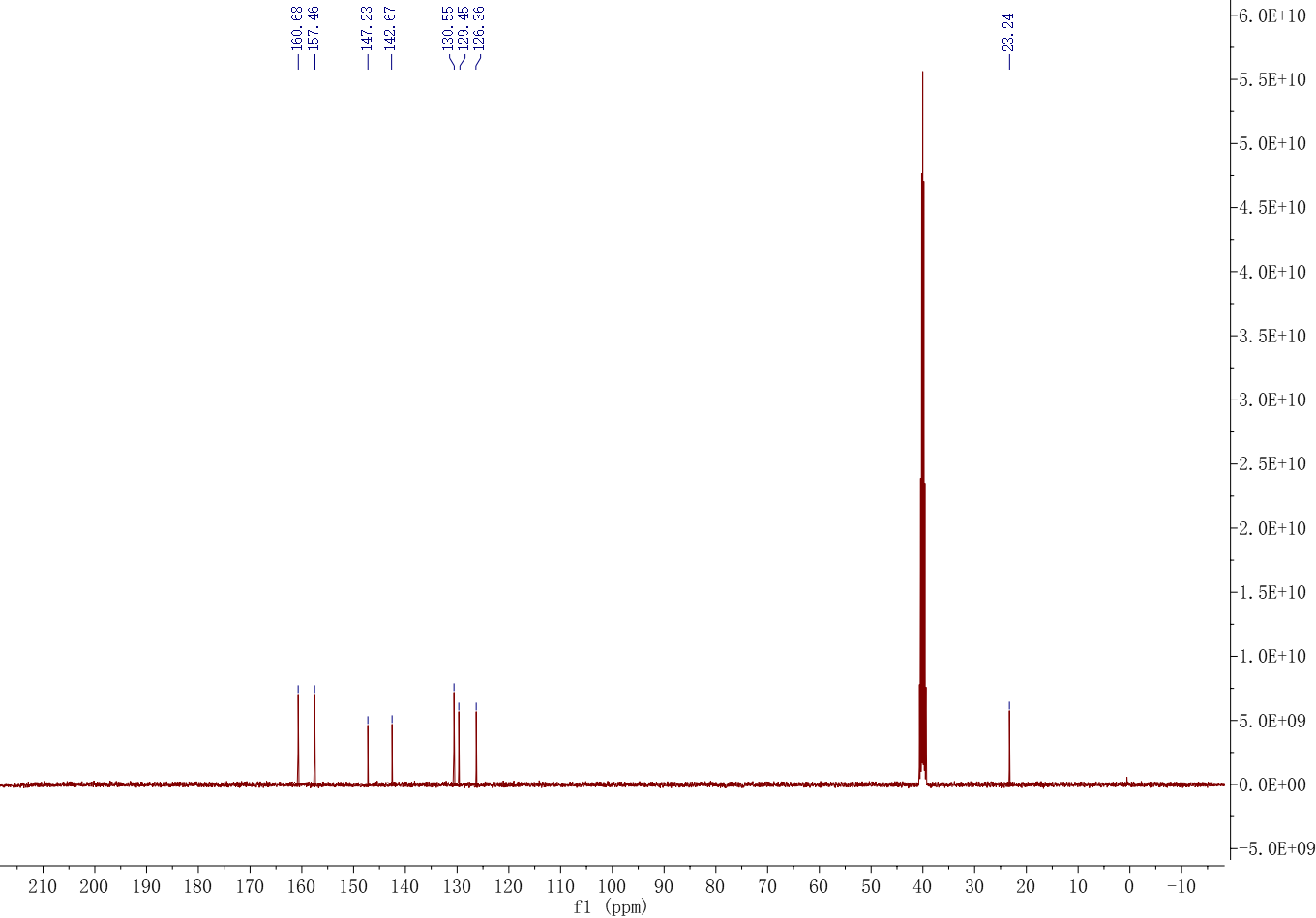


Fig. *^13^C NMR of* **E15** (100 MHz, DMSO)


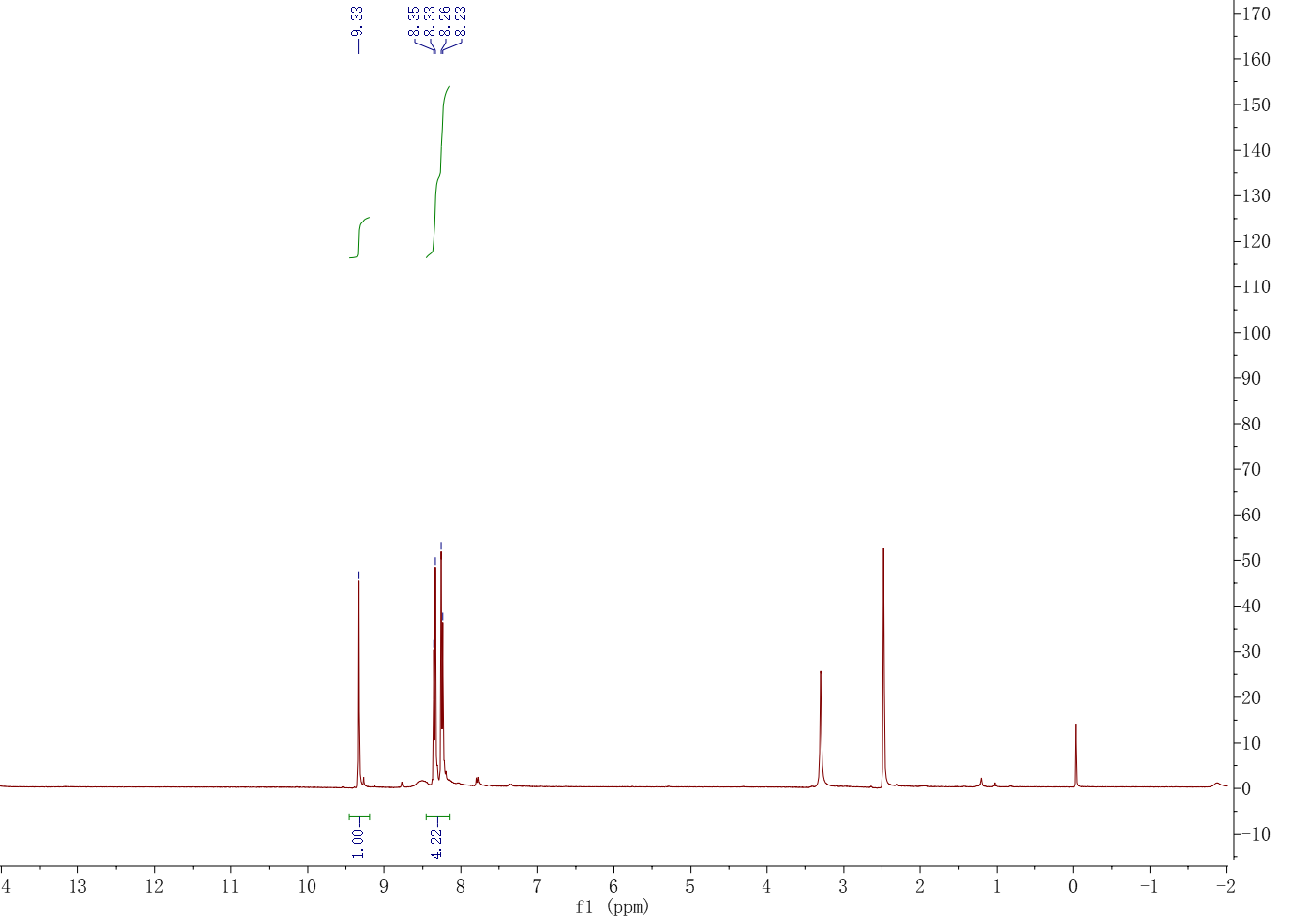


Fig. *^1^H NMR of* **E16** (400 MHz, DMSO)


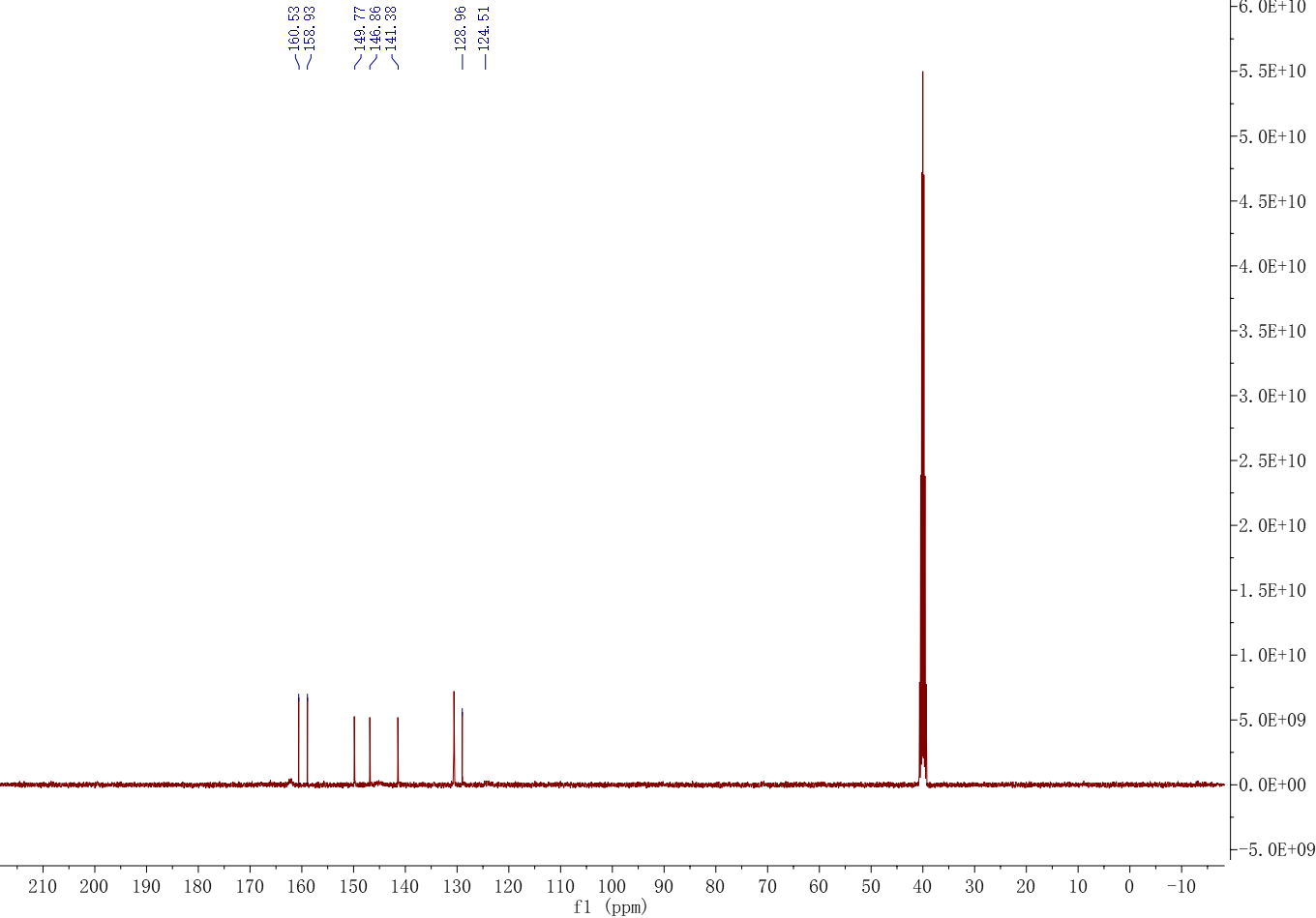


Fig. *^13^C NMR of* **E16** (100 MHz, DMSO)


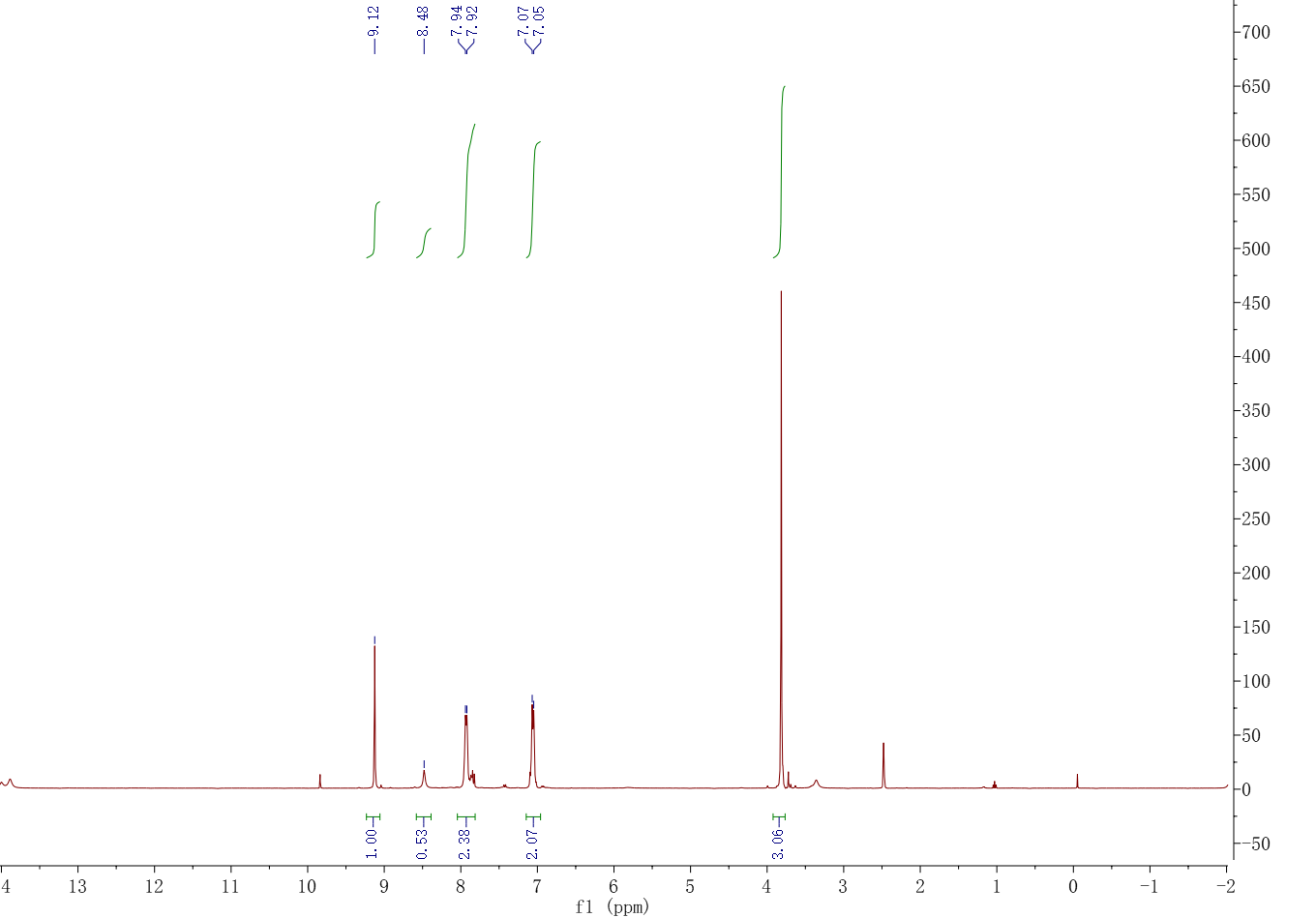


Fig. *^1^H NMR of* **E17** (400 MHz, DMSO)


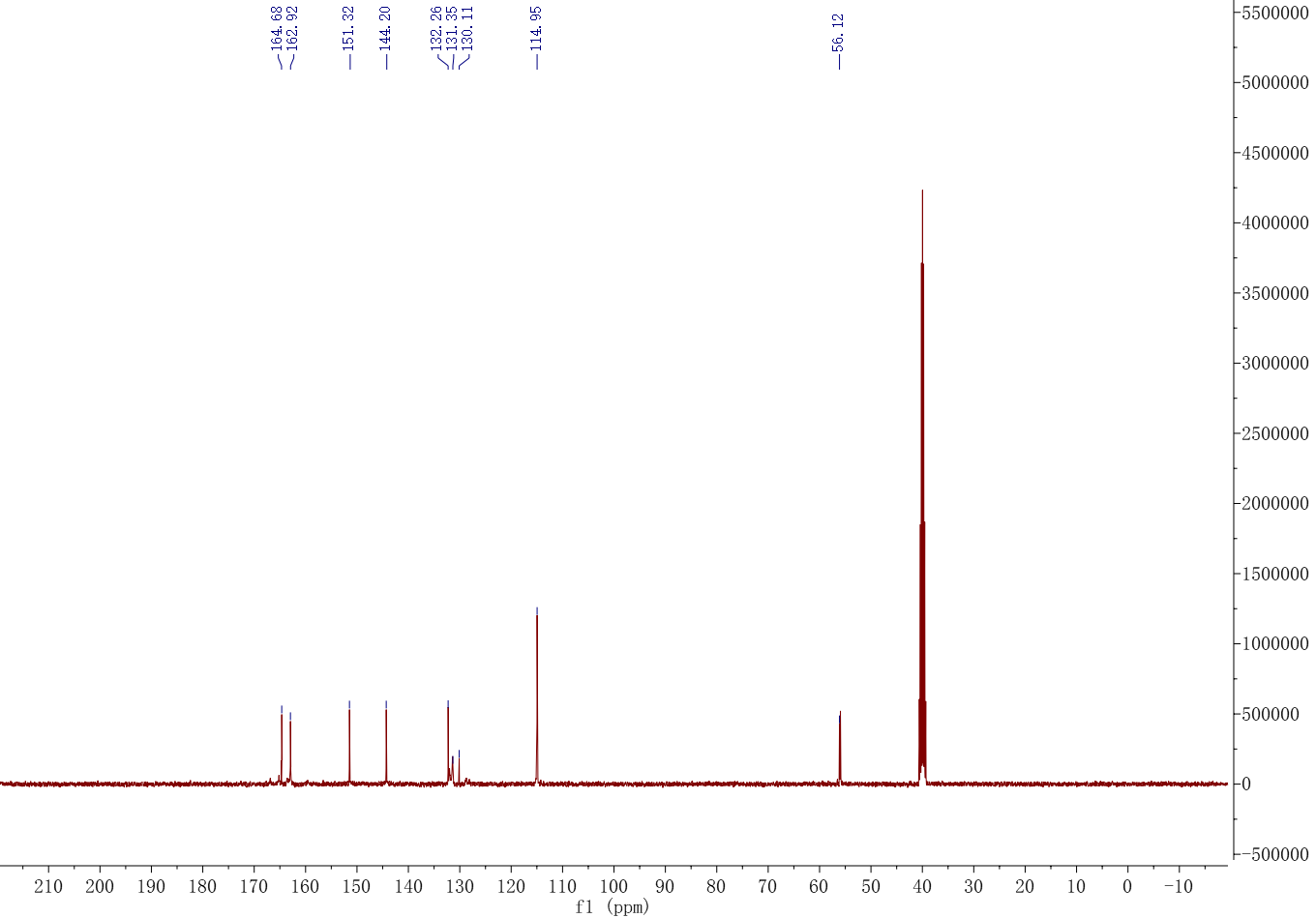


Fig. *^13^C NMR of* **E17** (100 MHz, DMSO)


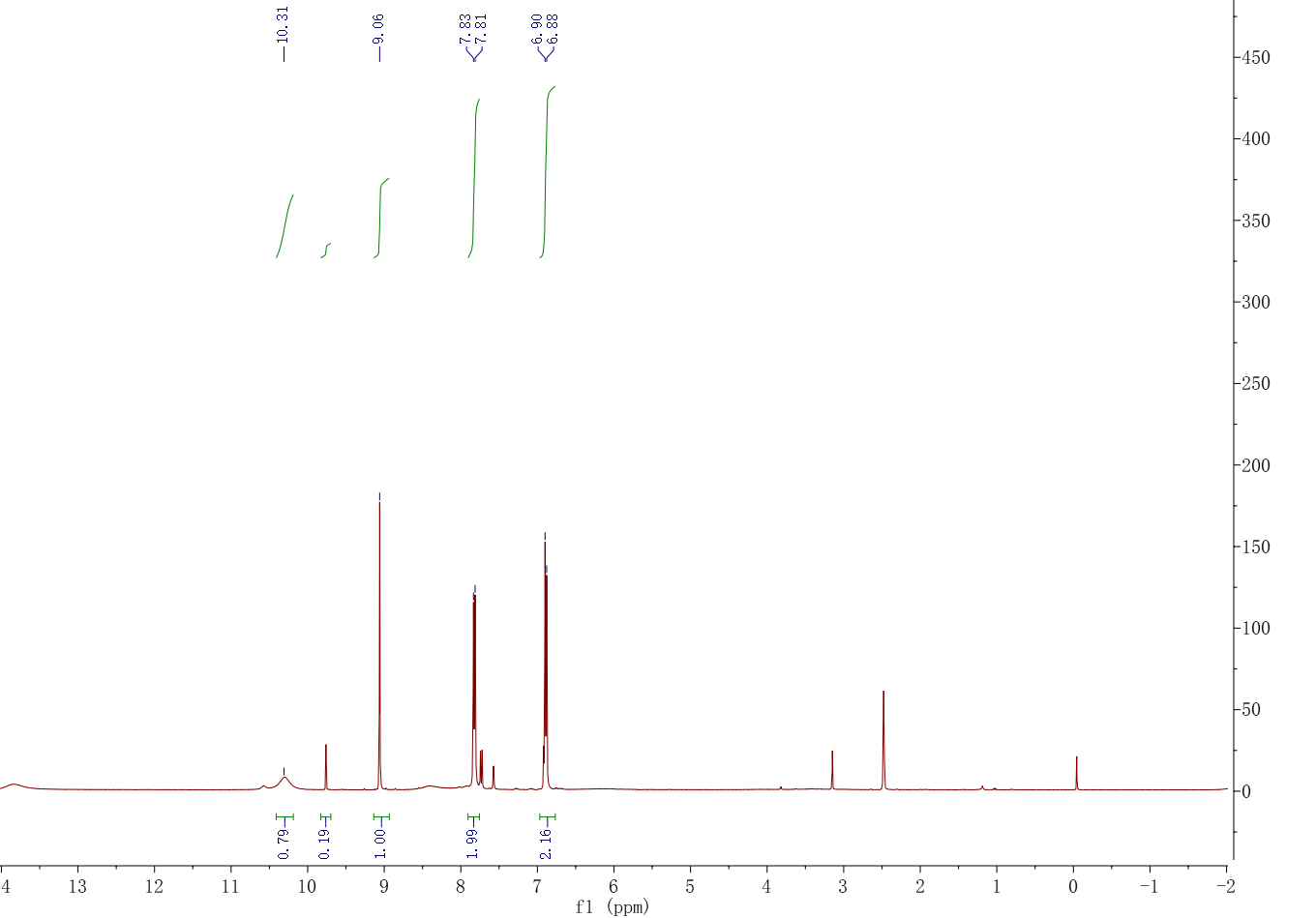


Fig. *^1^H NMR of* **E18** (400 MHz, DMSO)


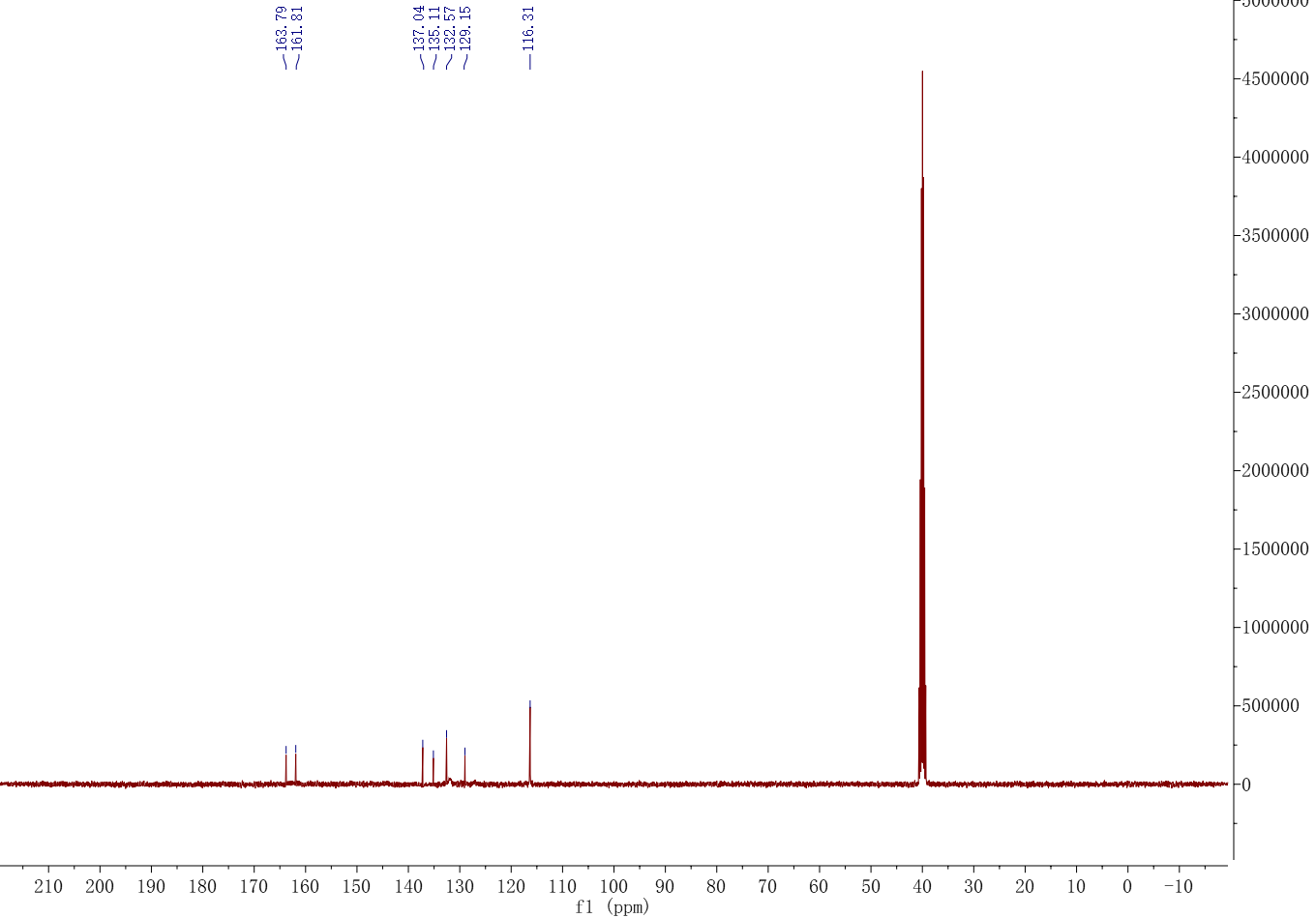


Fig. *^13^C NMR of* **E18** (100 MHz, DMSO)


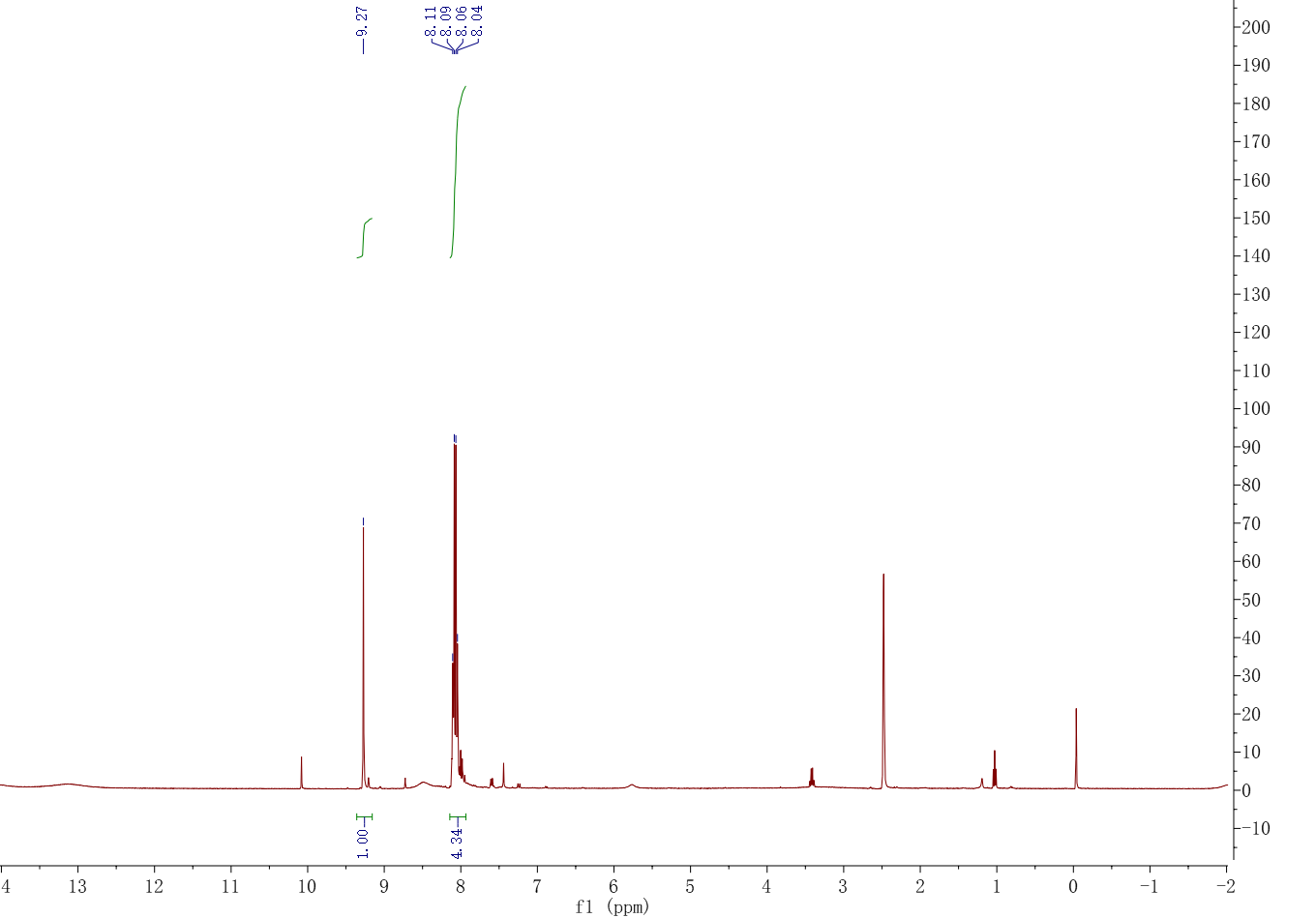


Fig. *^1^H NMR of* **E19** (400 MHz, DMSO)


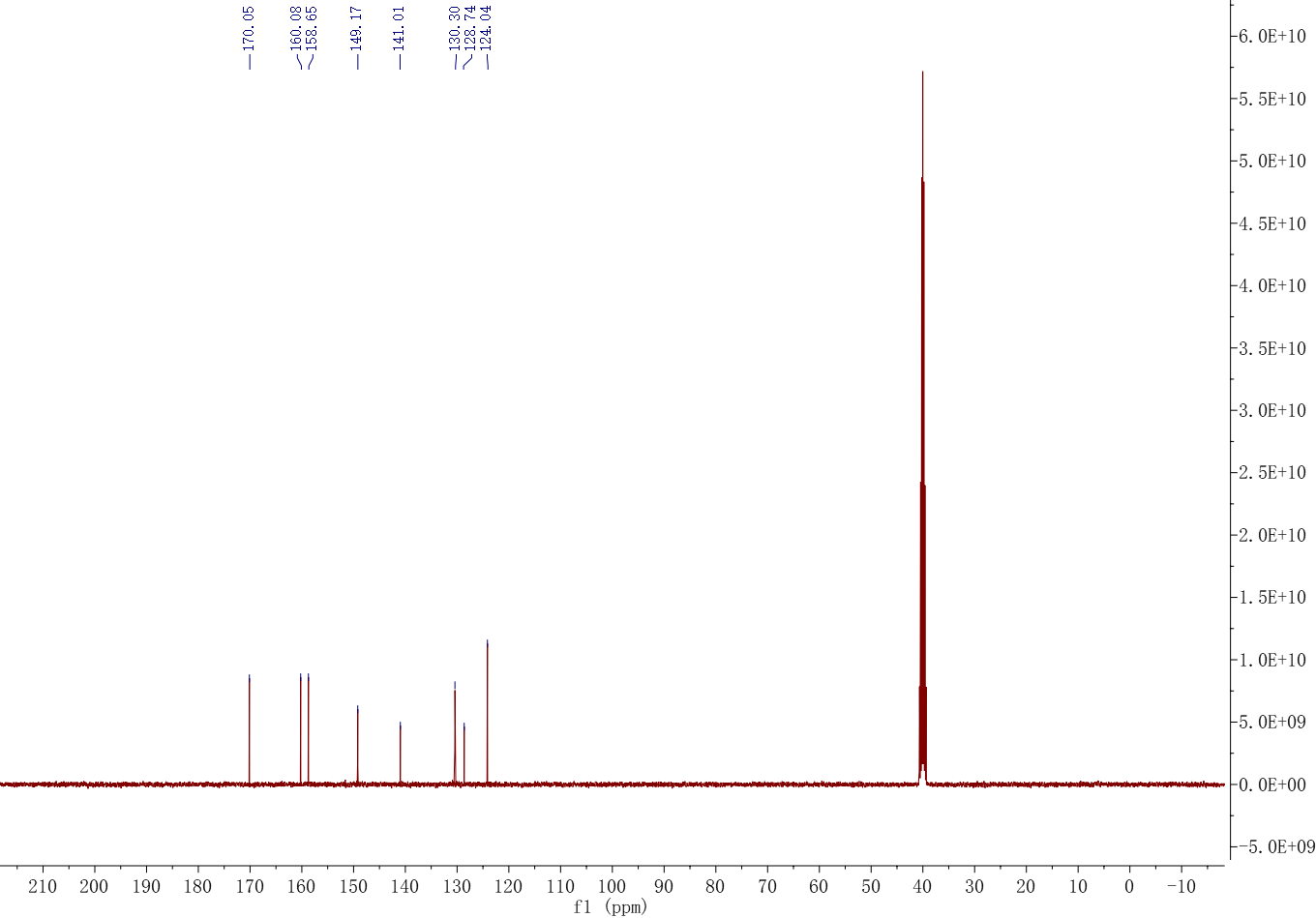


Fig. *^13^C NMR of* **E19** (100 MHz, DMSO)

**Fig**. *Mass spectrum of compound* ***E10***

1. *Correspondent. E-mail: [luhongzhi17@126.com](mailto:luhongzhi17@126.com) [↑](#footnote-ref-2)
